# Schistosomiasis and water resources development in Africa: A scoping review and multi-case evaluation of associated snail control

**DOI:** 10.1371/journal.pntd.0013180

**Published:** 2025-06-12

**Authors:** May N. Sule, Ibrahim El Lahham, Mutinta N. Munkombwe, Patricia Nasike, Anouk Gouvras, David Rollinson, Rashid Mbaziira, Comfort Kanshio, Giulio A. De Leo

**Affiliations:** 1 Centre for Water, Environment and Development, Cranfield University, Cranfield, United Kingdom; 2 Global Schistosomiasis Alliance, London, United Kingdom; 3 African Ministers’ Council on Water (AMCOW), Asokoro Abuja, Nigeria; 4 Department of Oceans and Woods Institute for the Environment, Hopkins Marine Station, Stanford University, Pacific Grove, California, United States of America; University of Heidelberg, GERMANY

## Abstract

**Background:**

Water resources development (WRD), specifically infrastructural man-made water bodies such as dams and irrigation schemes, are built to improve water supply, provide energy, and enhance food security. However, dams and irrigation schemes may lead to a dramatic increase in the prevalence of schistosomiasis.

**Methodology/Principal findings:**

We conducted a scoping review of WRD impacts on schistosomiasis transmission risk in Africa using electronic databases including Scopus, Web of Science, and grey literature. From 1483 retrieved records, we assessed 186 full-text papers and identified 122 articles covering 54 dams and irrigation schemes in 32 African countries. We found that, although the relationship between WRD and schistosomiasis transmission risk is well-documented in the scientific literature, the vast majority of the approximately 1,600 medium- to large-sized dams currently operating in endemic regions of Africa lack before-and-after prevalence data necessary to evaluate their actual impact on schistosomiasis transmission. Our analysis revealed that rigorous epidemiological data to assess WRD’s effects exist for only 11 dams across 9 countries. Additionally, only a limited number of studies provided information on schistosomiasis control methods, surveillance, or monitoring for WRD. Few countries have implemented engineering and biological snail control measures, some of which have proven effective, enabling us to identify successful interventions employed at various stages of the WRD lifecycle. Lastly, to assess these measures in detail, we selected case studies from Africa that illustrate the success and challenges of schistosomiasis control with regard to WRD, thus gaining insights of the global relevance of lessons learnt for the future development of water resources.

**Conclusions/Significance:**

Our analysis highlighted that an integrated and coordinated approach is vital for the successful control of schistosomiasis transmission risk associated with Water Resources Development. We provide key recommendations which could be adopted by the Continental Africa Water Investment Programme (AIP) with the ultimate goal of decreasing prevalence and moving towards elimination.

## Author summary

Man-made water bodies such as dams and irrigation schemes, are built to improve water supply, provide energy, and enhance food security. However, dams and irrigation schemes may lead to a dramatic increase in the prevalence of schistosomiasis (Bilharzia), a neglected tropical disease (NTD) with water snails as intermediate hosts. Schistosomiasis poses a significant public health threat in tropical and subtropical regions worldwide, particularly in low-income countries with inadequate water supply and poor sanitation. This scoping review and multi-case study assessed the impact on schistosomiasis following the construction of dams in Africa. Our results show that epidemiological data are available for only a tiny fraction of the ~ 1600 dams in operation in Africa. Data show that schistosomiasis does increase with the construction of dams but engineering and biological snail control measures, implemented in a few African countries, can reduce transmission risk. Example case studies further reflect on the successes and failures of implementation measures. We provided key recommendations which could be adopted by the Continental Africa Water Investment Programme (AIP) as part of the Programme for Infrastructure Development in Africa Priority Action Plan. Dams are earmarked to be part of the AIP delivery towards climate resilient water and sanitation investments by 2030. Political commitment, advocacy and multisectoral collaboration to support a health and environment integrated approach to dam development will be essential for achieving success.

## 1. Introduction

Schistosomiasis, also known as Bilharzia, is a neglected tropical disease (NTD) which poses a significant public health threat in tropical and subtropical regions worldwide, particularly in low-income countries [1]. With more than 250 million people affected in 78 countries and over 700 million at risk of infection, the vast majority in sub-Saharan Africa, schistosomiasis represents a significant health burden [2]. This parasitic disease, caused by trematode worms of the genus *Schistosoma,* is particularly prevalent in regions with inadequate water supply and poor sanitation, and is one of the most common NTDs worldwide [1].

The life cycle of the parasite typically involves two main hosts - humans and freshwater snails. The parasite develops in snails residing in freshwater (hereafter referred to as snail vectors). The most common species of snail that serve as hosts for schistosomes belong to the genera *Biomphalaria, Bulinus* and *Oncomelania*. Each transmits a different schistosome species: *Biomphalaria* hosts *Schistosoma mansoni* in Africa, the Caribbean and South America, *Bulinus* hosts *S. haematobium* in Africa and *Oncomelania* hosts *S. japonicum* in China, Philippines and Indonesia [2]. The snails have different habitat requirements and controlling snail populations is a key strategy in reducing the transmission of schistosomiasis. The larval stages of the parasite, the cercariae, exits the aquatic snail into freshwater and enter the bloodstream of the human host by penetrating the skin of individuals who come into contact with contaminated freshwater [2]. Once inside the human body, the schistosome parasites mature into adult worms and migrate to the veins surrounding the intestines or bladder causing intestinal (*S. mansoni, S. intercalatum, S. guineensis, S. japonicum, S. mekongi*) and urogenital (*S. haematobium*) schistosomiasis, with symptoms of painful and bloody defecation or urination respectively [3]. If left untreated schistosome infections cause immune reactions and lead to progressive organ damage [2].

Water resources infrastructure, specifically man-made water bodies, such as dams and irrigation schemes, have been built to improve water supply, provide energy, and enhance food security. There are presently ca. 1,600 dams in operation in Africa and over 300 are currently being constructed or are proposed [4]. This number does not include the numerous small reservoirs used for cattle and small-scale agriculture. The construction of dams creates an aquatic habitat that is generally suitable for the snail vectors to thrive, leading invariably to an explosion of snail populations [5,6].

In Africa, limited integrated approaches exist between sectors involved in water resources development and management, such as the water, agriculture and energy sectors [7]. The lack of coordination leads to the absence of, or lack of compliance with, or enforcement of, regulatory frameworks, social and health impact assessments. Because of the lack of coordination, the development of large dams has impacted sustainable economic wellbeing, resilience in communities, health and gender equality [8]. Globally, 13.6% of people at risk of schistosomiasis live in close proximity to irrigation schemes and large dam reservoirs [9]. Given the significant disease prevalence in Africa, it is crucial to understand the connection between the water, energy and agricultural sectors, so that regulatory actions and control measures are adopted for schistosomiasis when introducing water infrastructure projects. This is especially important because of the Continental Africa Water Investment Programme (AIP) which has been adopted as part of the Programme for Infrastructure Development in Africa Priority Action Plan by Heads of State and Government of the African Union (AU), comprising all 55 African member states. Dams are earmarked to be part of the AIP delivery, which is leveraging an additional $30 billion towards climate resilient water and sanitation investments by 2030.

The evidence of a link between dams and schistosomiasis was established following the meta-analysis by Steinmann et al., 2006 [9] and Sokolow et al., 2017 [10]. Both those reviews merged information about dams with datasets, such as the Global Neglected Tropical Disease Database GNTD, that jointly provided evidence of a before/after effect. However, most of those studies reported schistosomiasis prevalence data before dams’ construction, with studies that reported schistosomiasis prevalence data after dams’ construction, sometimes in different villages, schools or locations at variable distance from the dam. Hence, they were not designed as Before-After-Control-Impact (BACI) analyses. For the majority of the dams (~1200), this information is not available, generally because of the lack of baseline epidemiological data before the dam was built and also often because of the lack of good data after the construction of the dam. The majority of these types of water resources infrastructures are built without adequate epidemiological monitoring and surveillance plans in place for schistosomiasis. Perhaps this is because schistosomiasis has not often been considered a major issue within existing regulatory frameworks such as environmental and social impact assessments.

Hence, the objectives of this scoping review are: (i) to assess the impact of dams and irrigation schemes on schistosomiasis prevalence in humans across Africa and (ii) to assess whether engineering and biological snail control measures implemented at various stages of the lifecycle of water management infrastructures have been able to curb the negative health outcomes of water resources development. In addition, we provide a selection of recommendations drawing on lessons learnt from Africa and other global examples. This study will support efforts to enhance water resources management and public health outcomes in Africa and will help inform policy and decision-making at the national and regional levels.

## 2. Methodology

Our study protocol followed the PRISMA for Scoping Reviews (PRISMA-ScR) standards [11]. A comprehensive literature search was carried out from 6 March – 29 May 2023 to identify all relevant studies. The search was repeated from 20 – 21 October 2023 to identify any new relevant articles.

### 2.1. Search strategy

**Fig 1:**
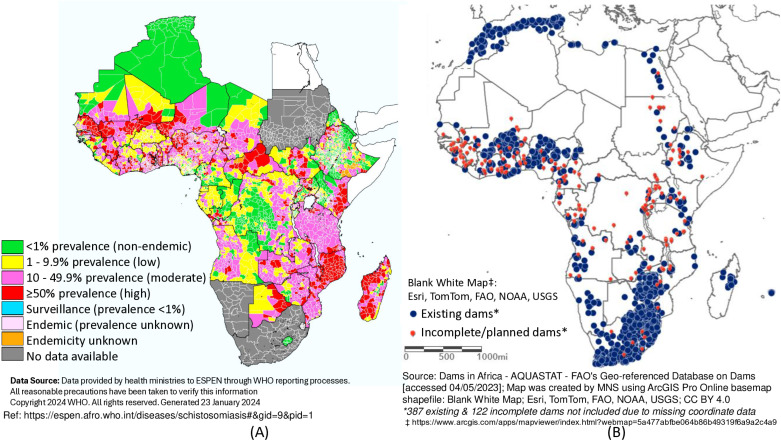
(A) Percentage range of people infected with Schistosomiasis, ESPEN-WHO [13] [accessed 09/05/2024]; the maps are updated annually and based on data sharing from countries; for countries indicated as “No data available” (e.g., South Africa) this is in reference to the indicated year, otherwise WHO has baseline data for those countries, and (B) Distribution of dams and reservoirs: FAO Geoportal database; survey data [4,14].

Firstly, African countries with dams and irrigation schemes were identified using the Food and Agriculture Organization of the United Nations’ (FAO) Global Information System on Water Resources and Agricultural Water Management (FAO AQUASTAT). Secondly, the countries identified were then matched with the World Health Organization’s (WHO) Expanded Special Project for the Elimination of Neglected Tropical Diseases (ESPEN) 2022 database of endemicity in prevalence of schistosomiasis across Africa, as shown in Fig 1. Through this process, we identified dams and irrigation schemes in African countries. A systematic search of studies relating to these identified dams, irrigation schemes and countries was conducted through searches of the following academic databases: Web of Science, Scopus and PubMed. The online databases of WHO, FAO and the World Bank were searched. Books and other grey literature such as dissertations were also considered [12].

The search terms used are shown in S1 Text. There were no temporal limits or language restrictions that were set for the search. The bibliographies of selected studies were also searched for relevant references and studies. Data collected were stored in Mendeley reference manager and exported to Microsoft Excel 2016 for data extraction and analysis.

### 2.2. Criteria for paper selection

At the title and abstract screening phase there were no restrictions on study designs or type. Peer-reviewed articles and grey literature were considered. Application of standard schistosomiasis control measures of mass drug administration (MDA)/chemotherapy with praziquantel, behavioral change, and health promotion campaigns were not considered an inclusion criteria, because they were outside the scope of this review. However, these measures were included in the recommendations as part of the overall integrated cross-sectoral approaches for schistosomiasis control.

Full-text articles were assessed for study quality and strength of evidence. We excluded papers of low quality as assessed by two reviewers using a 14-point quality appraisal framework (S2 Text). Factors used to identify suitable articles for inclusion were the availability of data, ascertaining the observed changes in schistosomiasis prevalence due to dams and irrigation schemes, and evaluating the adoption of engineering and biological snail control measures. Source selection, screening and extraction were performed by reviewers IEL, MNM, PN, and MNS, and any disagreements solved by consensus or by the decision of MNS.

Using these criteria, countries with comprehensive data were further selected for detailed evaluation of approaches and given priority over those with incomplete or inconsistent data. Hence, we selected six African countries Ghana, Senegal, Cameroon, Morocco, Côte d’Ivoire, and Egypt as case study examples of positive and negative outcomes associated with dams and irrigation canals for lessons learnt. The engineering and biological snail control measures implemented at design, construction, post construction and operational phases of dams and irrigation schemes in the identified countries over time, and their corresponding results, were combined to suggest key recommendations for implementation. These recommendations are aimed at informing policy and practice in water resources management and schistosomiasis control. Additionally, in separate general literature searches, three non-African countries – Brazil, China and Japan, were highlighted to draw on lessons from their demonstrated success towards schistosomiasis elimination as a public health problem defined as <1% prevalence of heavy intensity infections, or having achieved complete elimination of schistosomiasis infection.

## 3. Results

### 3.1. Characteristics of included African studies

**Fig 2:**
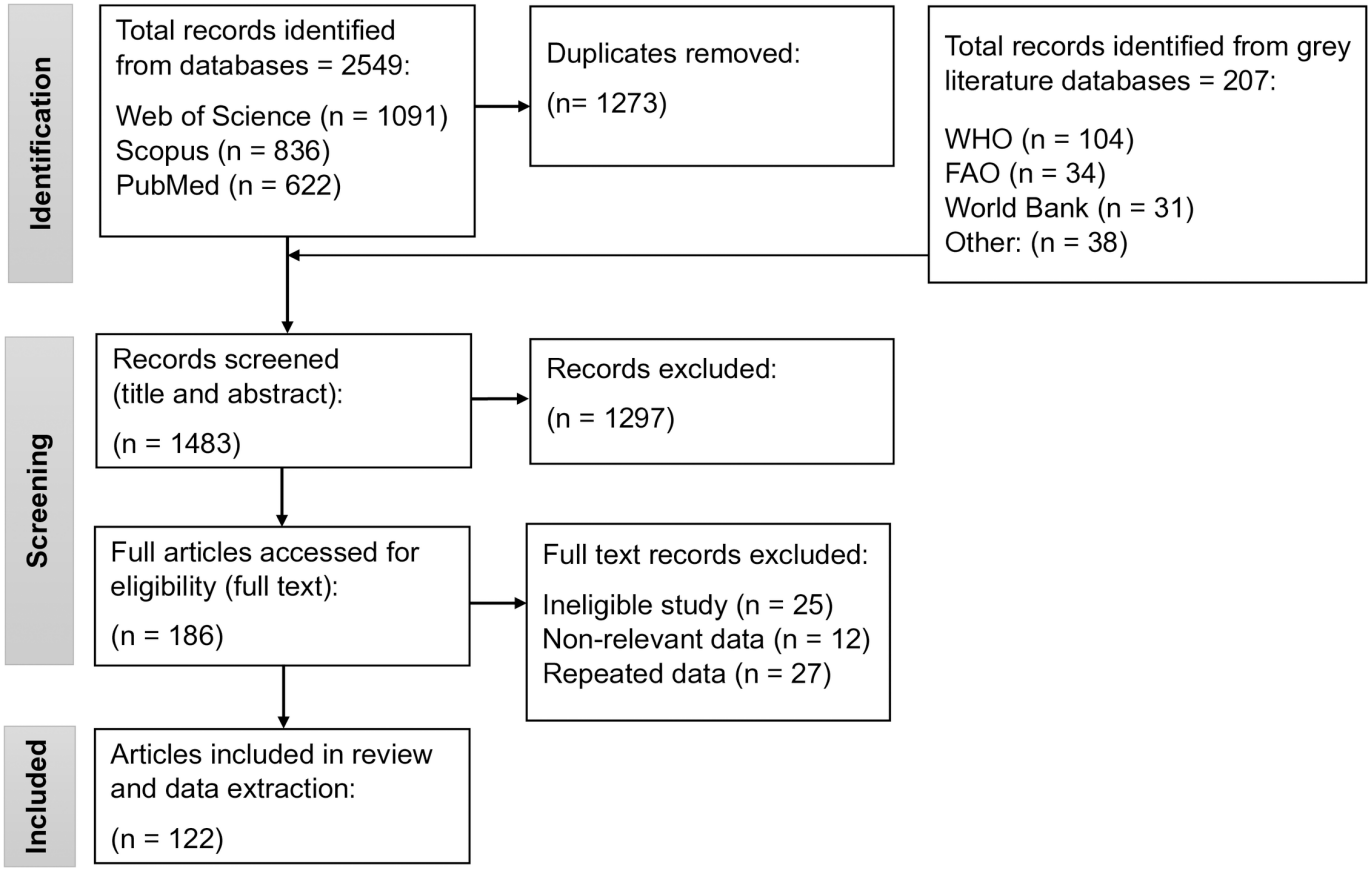
Flow diagram of article search and data extraction process for African countries.

All articles with relevant literature for 46 countries were identified and then those with data on percentage infection reported, percentage increase in schistosomiasis due to dam, engineering and biological snail control measures adopted during/before/after construction of the dam, and monitoring/evaluation were considered. From 1483 retrieved records, 1297 studies on application of standard schistosomiasis control measures of mass drug administration (MDA)/chemotherapy with praziquantel, behavioral change, and health promotion campaigns were excluded at title and abstract, and 186 full-text papers were assessed. There were 122 articles finally included in the comprehensive review as shown in the flow diagram in Fig 2.

### 3.2. Distribution of studies, evidence gaps and approaches

**Fig 3:**
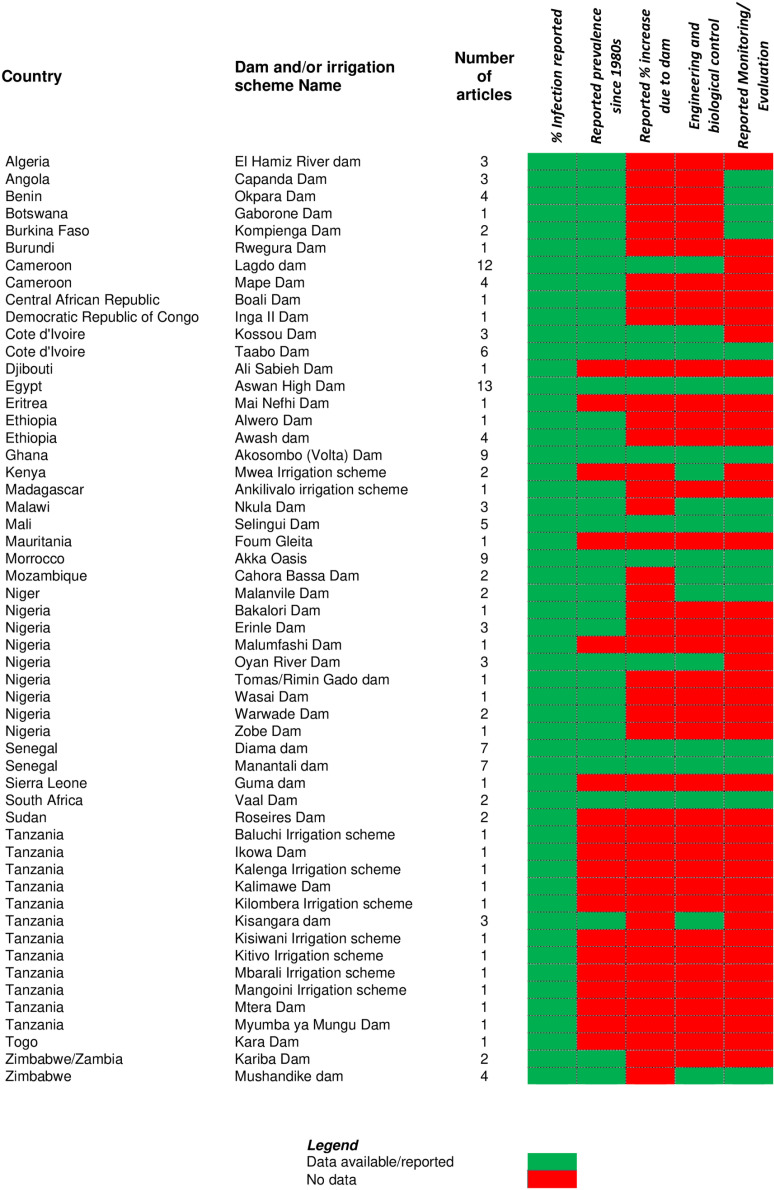
Heat map chart of dams and irrigation schemes with schistosomiasis occurrence in Africa included in the study with evidence gaps.

We found an uneven distribution of studies with many African dam and irrigation scheme locations having insufficient or no data availability. Prevalence data were reported for 54 dam and irrigation scheme locations in 32 countries included in the study with sufficient evidence highlighting the impact on schistosomiasis (Fig 3). Some information was available about the prevalence of schistosomiasis and the percentage increase in infection around areas surrounding the dams. Reported prevalence since the year 1980 was available for 35 (65%) dam locations. Information on the actual increase in schistosomiasis cases associated with dams and irrigation schemes (measured as incidence, changes in infection prevalence or after:before odd ratios) was available for only 11 (20%) locations. The remaining 43 (80%) locations had no information or demonstrated evidence of an association of increased transmission risk with the construction of the dam or the irrigation system, generally because of the lack of baseline assessment and/or systematic surveillance. The lack of baseline epidemiological data in many endemic settings hinders the establishment of a clear connection between increased prevalence and dam, reservoir and/or irrigation construction. Information on engineering and biological snail control measures adopted during, before and/or after construction of the dams were available for 17 (31%) dam locations and there was some information about surveillance or Monitoring and Evaluation being carried out in 16 (30%) dam locations. There were no recent studies carried out on the relationship of dams, irrigation schemes and schistosomiasis for some locations which limited this study to much older information in 19 (35%) dam locations. The impact of dams on schistosomiasis and the control interventions reported in the reviewed articles are provided in detail in S1 Table. Several categories of engineering and biological snail control measures were reported, as shown in S1 Table.

Our review identified further research gaps in the literature. It was difficult to determine the effectiveness of the interventions in reducing snail habitats, transmission and infection rates because of the lack of longitudinal surveillance and tracking data after such intervention measures were put in place. Some information was embedded in overall country data and therefore difficult to unpack or separate the effect of changes due to dams and irrigation schemes in those countries.

A detailed analysis of data from eligible sources was used to identify approaches for control measures. Table 1 outlines the various engineering infrastructure and biological snail control measures we identified which had been developed and implemented to reduce the risk of schistosomiasis transmission during the design, construction, post construction and operational phases of the dams and irrigation schemes along with description. Several actions had been taken to modify the environment around dams with the aim of affecting the natural habitat of snails. It was observed that some negative impacts of these snail control measures/actions could occur if engineering measures are poorly implemented.

**Table 1:** Implementation of engineering infrastructure and biological snail control measures at different phases.

Phase	Measures	Description and further notes	References
Design of the dam	Selected a site with minimal snail habitat	Schistosome snails prefer freshwater habitats that are shallow, slow-moving, and have aquatic vegetation. However, some species of schistosome snails can tolerate habitats that are deep, fast-flowing, saline, or polluted to some extent. For example, *Biomphalaria pfeifferi*, a host for *Schistosoma mansoni* in Africa, is an example of a schistosome snail that can survive in brackish water and sewage-polluted habitats. Schistosome snails require suitable substrates for attachment and egg-laying, such as mud, sand, rocks, or plants. Environmental factors such as temperature, rainfall, pH, dissolved oxygen, and water chemistry influence the distribution and abundance of schistosome snails	[15–18]
	Designed the dam to include adequate drainage	To further prevent the proliferation of snails and reduce the risk of transmission, it is essential to ensure that the dam has proper drainage systems in place. Installing drainage channels or pipes can direct water away from the dam and prevent the buildup of stagnant water where snails can thrive. A fish ladder is a structure on a river that allows fish to pass around a dam or other obstacle in the river. Restoring the migration of *Macrobrachium vollenhovenii*, the African river prawn, using a fish ladder can reduce the transmission of schistosomiasis. *M. vollenhovenii* preys on the snails that host schistosomiasis, effectively decreasing the spread of the disease.	[10,19–22]
	Planned for the installation of irrigation gates	Irrigation gates play an important role in promoting public health by controlling the spread of schistosomiasis. Irrigation gates help regulate water flow and prevent stagnation. Strategically placed gates can disrupt the life cycle of parasitic worms that cause schistosomiasis, reducing the risk of transmission.	[20]
During the construction of the dam	Constructed concrete banks to prevent snail colonization	Concrete lining and concrete banks can significantly impact the spread of schistosomiasis by reducing the availability of suitable snail habitats. Concrete lining prevents the buildup of snail-friendly environments, such as mud and algae, which are necessary for snails to survive and reproduce. This leads to a decrease in the overall snail population in the water, which in turn reduces the transmission of the flatworms responsible for causing schistosomiasis.	[23,24]
	Introduced natural predators	Introduction of certain fish species can help control schistosomiasis by preying on snails, which are intermediate hosts for the parasites causing the disease. Crayfish and carp are effective fish species as they consume snails and their eggs, preventing the snail population from increasing and breaking the life cycle of the parasites.	[22,25]
	Other Infrastructure development introduced	Improve access to water supply and safely managed universal sanitation coverage. Construct bridges over upstream and downstream rivers.	[24,26,27]
Post construction	Dredging practiced	Dredging the channels that branch out from the dam basin is an effective way to reduce the risk of schistosomiasis. Improved water flow and reduced stagnant water bodies through dredging can help eliminate the habitat where the parasite causing schistosomiasis can thrive. Dredging also helps to cover aquatic weeds growing on the banks of communities, which can eradicate intermediate host snails. The implementation of dredging can result in a significant reduction in the incidence of schistosomiasis and improved health outcomes in affected communities.	[28]
Operation	Ongoing monitoring and maintenance	Surveillance: Continue to monitor the dam and its surrounding areas for snail populations and any signs of damage or erosion. Conduct regular maintenance of the dam and its components to ensure that they are functioning optimally.	[29,30]
	Encouraged reduction of the risk of schistosomiasis transmission	Encourage the use of protective clothing and equipment when using water for domestic or agricultural purposes, as well as avoiding high-risk areas, such as snail habitats or areas with contaminated water.	[29,31]
	Water flow regulation	The “land-summer and water-winter” cycle is effective in controlling schistosomiasis, as it is contrary to the reproductive pattern of snails, which leads to a reduction in their population. This approach reduces flooding and snail density in marshlands, decreasing disease transmission	[32]

### 3.3. Selected case studies of effective measures and negative outcomes associated with dams and irrigation schemes on schistosomiasis in Africa

#### 3.3.1. Egypt.

Schistosomiasis in Egypt is largely attributed to irrigation canals and dams which provide suitable habitats for snails [33,34]. Until the 1930s, there was a high prevalence of urogenital schistosomiasis, at 60%, in the Nile Delta and Nile Valley regions, south of Cairo, where the Nile River flows past the Aswan High Dam and into districts with perennial irrigation [21]. However, the construction of the Aswan High Dam in 1964 resulted in a significant decline in the prevalence of urogenital schistosomiasis due to water flow regulation, which reduced snail habitats and in turn the transmission potential [35]. In contrast, intestinal schistosomiasis may have increased, both in terms of prevalence and distribution, after the construction of the dam in 1964 and until 1983. This increase can be attributed to the expansion of irrigation canals and drainage systems that favoured the snail hosts responsible for transmitting intestinal schistosomiasis [33]. Baseline levels of prevalence varying from 2 - 11% rose to 44–75% following dam expansion [36].

**Fig 4:**
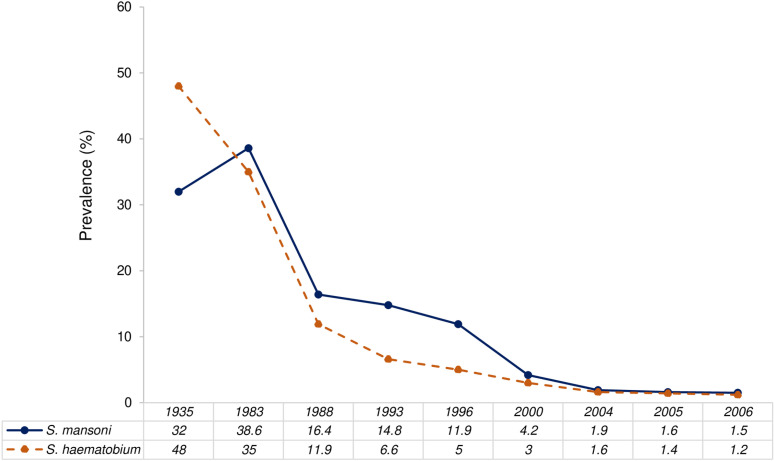
Overall prevalence of schistosomiasis in Egypt during the period 1935-2006 (Source: the National Schistosomiasis Control Program, quoted from WHO).

The national prevalence of intestinal schistosomiasis slightly increased from 32% to 39% between 1935 and 1983 [36]. However, Cline et al., 1989 [37] contested the accuracy of the 39% prevalence rate measured by Michelson et al., 1993 [36], suggesting that alternative testing methods could have yielded different results, including a potential decrease in the prevalence. Regardless, intestinal schistosomiasis steadily decreased after 1983 and until 2006 due to the integrated schistosomiasis control measures as shown in Fig 4 [37]. As a result, the prevalence of both types of schistosomiases decreased dramatically from 40% in 1967 to ≤ 3% in 2012 [38]. Currently, Egypt’s control program covers the entire infected population, while less than 1% of the total population requires preventative chemotherapy [39].

**3.3.1.1. Aswan High Dam water flow regulation:** The Aswan High Dam, completed in 1967, had a major impact on the Nile River’s ecology and irrigation patterns, leading experts to anticipate a higher incidence of schistosomiasis in Egypt [40]. However, studies have shown that there was a subsequent reduction in both intestinal and urogenital prevalence which was partially because there was also an on-going effort to control the snail population, which was a confounding factor of the net effect of the dam [33]. Furthermore, the dam’s regulation of water flow effectively reduced the transmission of urogenital schistosomiasis by preventing the formation of seasonal lakes, completely eliminating the snail habitat [21].

On the other hand, although the construction of the dam had a significant impact on disrupting the habitat of snails, it also resulted in an increase in water salinity and water logging, ultimately leading to a decline in soil quality and crop yields [35]. The construction of the dam also caused a deviation from the original course of the Nile River, resulting in erosion in the Nile Delta [41].

**3.3.1.2. Drainage projects:** The construction of drainage projects in the Nile River area has been a crucial strategy to prevent snail proliferation and reduce transmission risk in Egypt [21]. Drainage projects involved excavating canals and ditches to divert water away from snail habitats, disrupting their life cycle and reducing their populations effectively [42]. Although drainage projects have been proven to be effective in managing water flow and hence contributing to decreased snail populations, there are several potential drawbacks to consider. One significant disadvantage is the loss of land to the infrastructure required for an open drainage system [43]. Another potential concern is the extensive maintenance required to keep these types of drainage systems functioning properly [44].

**3.3.1.3. Concrete lining of channels:** Another method used by Egypt to control the transmission of schistosomiasis was to reduce the snail population in irrigation ditches. Two approaches used to achieve this were cement lining of ditches and the application of molluscicides [45]. The cement lining of ditches prevents snails from attaching and breeding, while molluscicides kill the snails. Although these techniques were successful in reducing the snail population, they have some drawbacks [46]. Canal lining can adversely affect groundwater supply, reducing the amount of water reaching the aquifer [47]. Additionally, installing and repairing the lining of the canal can be challenging and requires significant investment and maintenance costs [48].

**3.3.1.4. Introduction of crayfish and black carp:** During the early 1980s, the introduction of crayfish to the Nile Delta for aquaculture had unintended positive consequences in terms of reducing the snail population. The crayfish rapidly spread, became invasive, and were found to prey upon snails that transmit schistosomiasis, serving as a biological control agent [49]. Another biocontrol agent of snails in Egypt is the black carp, which feeds exclusively on snails. The introduction of both the black carp and crayfish led to a reduction in the biomass of aquatic plants and snails, resulting in a decrease in the population of the snails and consequently the transmission of schistosomiasis [49]. However, the introduction of these non-native species led to the displacement of native species along the Nile, which negatively impacted the biodiversity of the region [50]. Additionally, these invasive species compete with other native species for food, which can further disrupt the delicate balance of the ecosystem [51].

#### 3.3.2. Morocco.

Historically, schistosomiasis was not widely distributed in most parts of Morocco due to water scarcity. However, with the expansion of irrigation projects to arid parts of the country, the prevalence of the disease increased in the 1970s [31,52]. The number of cases detected and recorded during this time reached the highest peak of 13,416 in 1973. The construction of dams and canal systems provided favorable conditions for increased populations of *Bulinus truncatus*, the snail intermediate host. Consequently, as the canals became an important source of water for both agriculture and domestic use, there was an increased risk of exposure in areas which later became schistosomiasis foci [53].

**Fig 5:**
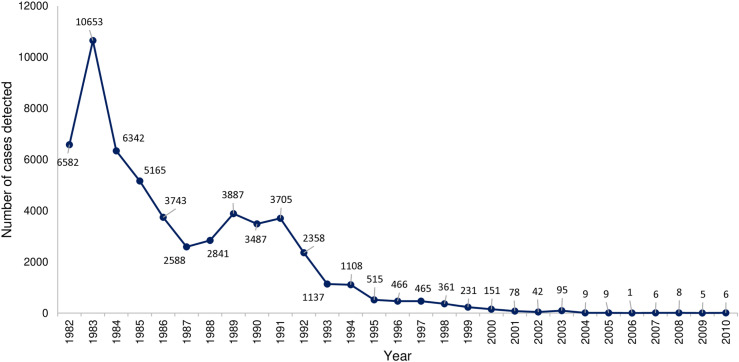
Decline in schistosomiasis cases, Morocco 1982 – 2010 (Sources: Annual Reports of the National Program against Schistosomiasis, Ministry of Health, [ 56]).

In response to the increasing prevalence, in 1976 Morocco developed a National Schistosomiasis Control Programme (NSCP), which became operational in all provinces by 1982 [53]. The program outlined an integrated approach to control and elimination through screening, treatment, snail transmission control, health education and community participation. This approach was a success and as of 2004, no indigenous cases of schistosomiasis were reported [54]. An interruption of transmission was confirmed by a serological study of disease-endemic foci whereby results showed an absence of antibodies in all serum samples [55]. Following the commencement of the NSCP, the total number of cases detected declined from 10,653 in 1983–6 in 2010, as shown in Fig 5 [52,56].

**3.3.2.1. Mechanical methods:** Mechanical measures to control snails involved modifying the snail biotope or habitat to prevent their proliferation. These methods included changing the physical environment of the snails, such as reducing the amount of stagnant water or eliminating vegetation that provides breeding ground for snails [29,30]. This was achieved by dredging or draining water bodies, removing aquatic vegetation, or excavating silt. Another mechanical method was the brushing of edges of the sumps after each irrigation exercise. Brushing the edges of sumps disrupted the snail habitat and dislodged snails. Periodic maintenance of various network structures was also used as a mechanical method of snail control [29,57]. This involved repairing or replacing broken pipes, valves, or other equipment that can cause water stagnation, which favours snail breeding.

The use of mechanical measures to control snails may be costly and time-consuming to implement, especially in large water bodies. For example, dredging or draining a large lake or river may require significant resources and equipment, which may not be available in some areas. Additionally, these methods may disrupt the natural ecosystem of the water body and impact other organisms that depend on snails and aquatic vegetation for their survival [51]. Moreover, mechanical methods may be temporary solutions and may require ongoing maintenance and monitoring to be effective. For example, brushing the edges of the sumps may remove snails, but if the snail habitat is not permanently disrupted, they may return to the area and re-establish their population.

**3.3.2.2. Environmental management:** Emphasis was also placed on reducing the population of the snail intermediate host in irrigation systems. Tertiary canals and siphon boxes were particularly important habitats for snails as they provided favorable conditions for breeding [58]. Focal and gravity mollusciciding through the routine application of niclosamide was used as the main snail control measure in several irrigation schemes. Given the cost and potential effects on non-target organisms of mollusciciding, alternative snail control measures were attempted alongside mollusciciding in the Tessaout- Amont irrigation scheme [59,60]. These included placing concrete covers on syphon boxes to minimize light entry and brushing the sides of syphon boxes. All these methods were shown to reduce the snail population over the study duration period, however, periodic mollusciciding was evaluated as the most effective method to prevent snail repopulation [60].

Community engagement also proved beneficial in the reduction of snail populations through environmental control. In the Akka Oasis in South Morocco, the local irrigation committee conducted routine cleaning and clearing of vegetation in canals and impoundments on the Akka riverbed. This practice yielded tangible results in the reduction of snail and egg mass densities in the area [29].

#### 3.3.3. Ghana.

Schistosomiasis had a high prevalence in Ghana, at 71% as of 2010 [61,62]. The presence of urogenital schistosomiasis has been identified in all regions, with varying levels of prevalence ranging from less than 10% to over 70%, with the western regions exhibiting the highest prevalence rates [63]. Prevalence rates can be as high as 80–90% in communities located along the lakeshores of the Volta basin, while the Volta estuary is also endemic, with infection rates of 76.2% for intestinal and 6.3% for urogenital schistosomiasis [62,64]. The construction of Lake Volta in the 1960s, as well the construction of Akosombo and Kpong Dams, significantly increased the incidence of schistosomiasis prevalence across Ghana, heavily infecting all regions [65]. Despite some regions exhibiting lower prevalence rates, the disease continues to pose a significant public health challenge in the country, particularly in areas where water contact is frequent.

While data published in the Global Burden of Disease (GBD) platform in 2023 demonstrated overall reductions in prevalence, population increases in many endemic areas have sustained or even increased the number of individuals considered at risk for schistosomiasis [66]. Despite these challenges, the prevalence of schistosomiasis in Ghana has decreased by approximately one-third since 2000, indicating the effectiveness of the various control measures implemented in the country [65].

**3.3.3.1. Environmental improvement projects:** Since the 1960s, interventions were initiated to control schistosomiasis in villages surrounding Lake Volta [67]. The various methods implemented, such as aquatic weed clearing, vector snail surveying and sampling, construction of low-maintenance pit latrines, and health education, had a significant impact on the transmission and control. These interventions helped reduce the abundance of snail populations, identified areas with high snail populations for targeted intervention and assessed the effectiveness of existing control strategies [67]. In some of the villages where these methods were implemented, there was evidence of decline in schistosomiasis incidences. However, despite this decline, the occurrence of the disease remained relatively high compared to the situation before the creation of the dam [68].

While the aquatic weed clearing can be effective in controlling schistosomiasis, its implementation can also have potential negative impacts such as unintended environmental disruption, resource-intensive and challenging implementation. For example, aquatic weed removals led to loss of habitats for some aquatic life and species (fish, plants etc.) as well as contributing to soil erosion in areas around dams and canals in Ghana [69].

**3.3.3.2. Saltwater intrusion and dredging in Ghana:** The construction of the Akosombo and Kpong Dams reduced river discharge and caused the formation of a sandbar, which prevented saline seawater intrusion upstream during high tides, creating a favorable snail habitat [70]. As a result, there was an increase in the growth of vector snails, which increased the incidence of schistosomiasis infections in the surrounding communities [71]. To address this, the Volta River Authority (VRA) dredged through the sandbar and deepened the silted channels to restore saltwater intrusion [28]. Dredging operations interconnected deeper ponds, allowing saltwater to penetrate the whole tidal area, and served as a protection for communities inhabiting the lower banks from flooding. Dredging also covered aquatic weeds growing on the banks of the communities, helping to eliminate snails. Dredging activities were beneficial in preventing floods and maintaining a salinity level to counteract snail colonies up to 9 km from the estuary [72]. As a result, the incidence of schistosomiasis has decreased significantly in recent years. Nevertheless, a major obstacle is insufficient equipment for the operations [28].

Dredging and saltwater intrusion pose a significant threat to the environment, with potential long-term effects that can be detrimental. These activities may impact on topsoil fertility, destroy essential fish spawning areas, making riverbanks unstable and vulnerable to erosion [73]. In turn, this can lead to habitat loss for riverbank animals and negatively impact local fish populations [74]. Saltwater intrusion can also have a dreaded consequence of negative impact on human health [75].

#### 3.3.4. Cameroon.

Schistosomiasis distribution is uneven in Cameroon, ranging from 1.7%-55.5%, with a higher prevalence in the northern regions [76,77]. The development of dams for hydroelectric power and irrigation canals, along with a lack of potable drinking water has led to a high prevalence in the country. The dams and irrigation schemes created optimal conditions for the multiplication of snails and consequently, the spread of schistosomiasis [10,78]. Mape dam increased schistosomiasis outbreaks.

In northern Cameroon, the distribution and hotspots of schistosomiasis are linked to proximity to Lake Chad and the Benue River, on which Lagdo dam is located [9,61]. The construction of Lagdo dam in 1982 created a 700-square-kilometre artificial lake, which is a reservoir for irrigation, consisting of a 3-kilometre open unlined main canal that divides into two concrete-lined principal canals, creating favorable snail breeding conditions [79]. In the area around Lagdo dam, the prevalence of schistosomiasis was at 15% in 1968, which then increased to 61% by 2000. Similarly, at the national level, the schistosomiasis situation worsened around 1987, when the first irrigation scheme was constructed. More specifically, the prevalence rose from between 7–21% in 1986 to 43% by 1992 [80]. These statistics suggest that the dams and irrigation projects may have contributed to the increase in the schistosomiasis prevalence. However, it is worth noting that some researchers attributed the increase in the early 1980’s to the country’s failure to develop a national strategy for the control of schistosomiasis [61,81].

##### 3.3.4.1. Poor water flow management:

Lagdo dam led to the creation of a large reservoir that allowed for the development of an extensive irrigation project covering thousands of hectares [82]. However, an unintended consequence of the project was the transformation of natural floodplain depressions into marshy areas [83]. These marshy areas provided ideal breeding grounds for freshwater snails, including some intermediate host species of schistosomiasis. Combined with the development of irrigation projects that lacked snail control measures, ideal snail breeding conditions were created, leading to an increase in the prevalence of schistosomiasis [83].

##### 3.3.4.2. Absence of bridges:

The villages located in the vicinity of Lagdo dam are situated on a low terrace, bordered by a river and a depression. People frequently walked through the depression to access the terrace, despite the fact that it is continuously inundated with water [83]. This geographical feature has negatively impacted the health of villagers around both Lagdo and Mape dams as the enhanced contact with the water has been linked to a higher prevalence of schistosomiasis [84,85].

#### 3.3.5. Côte d’Ivoire.

The prevalence of schistosomiasis in Côte d’Ivoire varies from as low as 1% to as high as 90% in different parts of the country [86–88] with high prevalence among school aged children living in close proximity to dams and irrigation schemes [89].

Kossou and Taabo are two of the largest dams, located along the Bandama River in central Côte d’Ivoire. Both were built in the 1970s to provide hydropower generation, irrigation, flood control and water supply. They are classified as embankment dams and are built with earth and rockfill [90]. A study revealed that the Kossou dam had a leakage problem at its base, providing a favorable habitat for snails [91]. As a result, the prevalence of *S. haematobium* increased from 14% to 53% around Lake Kossou. Similarly, the construction of the Taabo dam around Lake Taabo led to a surge in *S. haematobium* prevalence from 0% to 73%. Additionally, although the reservoir banks are steep, water in the dam covered vegetation that existed before dam construction, providing an ideal food base for snails [91].

**3.3.5.1. Lack of water and sanitation infrastructure:** The lack of adequate water, sanitation, and hygiene (WASH) facilities in Côte d’Ivoire posed a significant risk of infection to the local population [92]. Open defecation has also been linked to a higher prevalence of schistosomiasis in Côte d’Ivoire [86]. In addition, heavy rains and flood events exacerbated the situation in endemic areas. Poorly maintained sanitation infrastructure resulted in the overflow of sewage and waste, leading to the contamination of water sources and increased coverage of schistosome-carrying snails [93]. Furthermore, activities, such as agriculture, fishing, and washing clothes, are often conducted around man-made dams and water sources, which further increases the risk of transmission [86].

**3.3.5.2. Haphazard and unsystematic construction of dams:** The construction of dams in Côte d’Ivoire has not followed a planned or organized approach. A total of 22 dams over 10m high were built mainly in the 1960s and 1970s without relevant regulations or guidelines, resulting in a lack of consideration for potential environmental and health impacts [94]. The impacts were due to several factors, including the creation of new water sources, alteration of water flow, extensive population movements, increased agricultural activities, and human contact with infected water [91].

#### 3.3.6. Senegal.

Schistosomiasis prevalence in Senegal has been affected by the ecological changes that have taken place in the Senegal River Basin (SRB) [95,96]. These changes are the result of two major construction projects: the construction of a dam at Diama on the Senegal River in 1986, which was built to prevent sea water from intruding into the river, and the construction of a dam at Manantali, Mali on the Senegal River Basin, which was built to regulate water flow and generate electricity [15]. Prior to the construction, there was low urogenital schistosomiasis prevalence in the region and intestinal schistosomiasis was not endemic. Following the completion of the Diama dam, there was a massive increase in schistosomiasis incidences. Prevalence of infections in school aged children ranged from 43% to 99% for urogenital schistosomiasis and between 2% and 95% for intestinal schistosomiasis, where transmission occurred all year round [97]. An outbreak of intestinal schistosomiasis occurred in the SRB, with a high prevalence of up to 100% in some local populations [96,98,99], making the SRB one of the most schistosomiasis hyperendemic regions of the world. More recent data show some reduction in intestinal schistosomiasis prevalence in the lower basin of the Senegal river with urogenital schistosomiasis becoming the dominant infection despite repeated rounds of mass drug administration in research settings and through the national control program [100,101]. On a small scale, manual removal from transmission sites of aquatic vegetation where snails thrive is effective in reducing schistosomiasis transmission. The harvested vegetation can be used to produce compost for agriculture and biogas or integrated in animal feeds for livestock, a win-win for human health and the fight against poverty and malnutrition that targets multiple SDGs at once [100].

**3.3.6.1. Poor dam design:** The construction of dams and irrigation schemes that alter the ecology and hydrology of the Senegal River basin by preventing saltwater intrusion played a major role in increasing the population of snails [95]. For example, in 1988, the construction of the Diama dam caused the elimination of saline water conditions at the mouth of the Senegal river, which was the intended goal of the dam. Permanent freshwater upstream from the dam and the increased use of agrochemicals associated to agricultural expansion and intensification have created favorable conditions for the proliferation of the aquatic vegetation that is suitable habitat for schistosome parasite-competent snails [10].

**3.3.6.2. Elimination of natural predators:** Once the Diama dam was finished in 1986, it prevented the yearly migration of river prawns which feed on snails [102]. Before the construction of the dam, when river prawns were plentiful, cases of human schistosomiasis were rare [103]. However, the dam now obstructs the migration of female prawns downstream to the estuary and prevents the upstream movement of larvae, leading to the decrease in prawn population [102]. This decline in the prawn population above the dam coincided with a significant increase in the prevalence of human schistosomiasis in the Lower Senegal River Basin [10,22].

### 3.4. Examples outside Africa

*S. mansoni* is endemic in central- and north-eastern Brazil. Here the prevalence of schistosomiasis infections decreased from 15.6% to 9.5% between 1950 and 1990 [104], thanks to increased surveillance and medical treatment, improved sanitation, and the construction of sewage treatment facilities [105]. Historically, schistosomiasis was largely endemic in three areas of Japan where the parasite *S. japonicum* infects snails of the genus *Oncomelania*, such as *Oncomelania hupensis* as intermediate host, and, in addition to humans, more than 30 mammals’ as definitive hosts, including several wildlife species as well as cattle, dogs, cats, rodents, pigs, horses, and goats [106]. However, Japan implemented a successful schistosomiasis control program that mainly focused on snail control. The last reported human case of schistosomiasis was in 1977, and the country declared elimination in 1996 [107,108]. Similarly, before the mid-1950s, China had one of the world’s worst schistosomiasis situations, with a high prevalence and intensity of infection caused by *Schistosoma japonicum* [109]. This parasite was endemic in 12 provinces and affected about 11.6 million people in 433 counties or cities in the mid-1950s [110]. The number of individuals infected has declined from 11.6 million in 1956 to just 0.3 million in 2015 [111]. China declared schistosomiasis to no longer be a public health concern in 2015 and is currently striving to halt transmission and eliminate the disease by 2030 [109].

The implementation of mechanized irrigation systems contributed significantly to the reduction of schistosomiasis prevalence in certain areas. For example, a study conducted in the State of Bahia, Brazil, found that the expansion of irrigation systems did not lead to an increase in urogenital schistosomiasis infections [112]. This was attributed to the adoption of advanced technologies that require minimum human contact in irrigation and agriculture, which reduced direct contact between farmers and water. Similarly, schistosomiasis elimination in Japan coincided with the development of mechanized agriculture, such as use of tractors instead of oxen and horses [113]. However, mechanized irrigation systems have several challenges, including high installation and maintenance costs, high energy requirements, potential soil erosion, and limited crop selection [114].

Environmental modifications were made to snail habitats through the widespread use of concrete in Japan [106]. Snail populations were known to be highest in rice field ditches and to counter this, ditches were lined with concrete [115]. Similarly, China used concrete lining in irrigation ditches, which involved covering the walls and floors of these ditches with concrete and they also modified marshlands [116]. This technique helped to prevent snails from living in the moist environment of the ditches, thus reducing the risk of disease transmission [117]. As of 2015, roughly 40% of wetlands in villages where the disease was common had been drained, and about 30% of irrigation ditches had been lined with concrete [118]. Cement lining in Japan disrupted the natural flow of water, destroyed habitats, reduced the availability of nutrients for rice cultivation, increased the risk of flooding, and caused soil erosion [119]. It was disruptive to the natural habitats of species living along the banks [120]. The use of concrete lining can also be expensive to maintain [121].

Between 1985 and 1995, Brazil decreased the prevalence of schistosomiasis through the implementation of small dams. These dams were used to raise tilapia fish, which served as biological control for snails [122]. As a result of the project, the prevalence of schistosomiasis decreased significantly from 30.9% to 4.3%, and its intensity also decreased noticeably [123]. Tilapia tend to consume native plants like algae and water lilies, which can have negative consequences for other small animals that rely on these plants for survival [124]. Furthermore, tilapia can contribute to an increase in sediment in the water, which can hinder the growth of plants by blocking sunlight [125]. Japan also involved the introduction of snail predators such as geese or firefly larvae, although these measures showed no evidence of success [107]. The introduction of predators may not always be effective, and it can have unintended ecological consequences, particularly if the predators are not native to the region [107].

Changes in land use also had a significant impact on schistosomiasis control in Japan. Paddy fields were converted to orchards and residential areas, changing the economy and social structure in these areas [126]. The change in land use also saw the construction of social amenities, such as communal swimming pools, thereby limiting exposure to natural water bodies [107]. Agricultural practices were also transformed to prevent disease spread. Horses were recommended to replace cows as labor animals after studies showed that they were more resistant to schistosomiasis than cows. Night soil, commonly applied directly to rice plantations as a fertilizer, was stored for at least two weeks before use, killing the schistosome eggs [108]. Among fertilizers’, caustic lime was found to have a killing effect. Environmental degradation caused by changes in land use can lead to soil erosion, loss of biodiversity, and degradation of water quality.

The Three Gorges Dam situated in the middle of the Yangtze River in China has an important role in flood control and regulating water flow. The dam changed the water levels spatial and temporal variability, leading to a significant impact on the ecology of the region and the spread of schistosomiasis [127]. The dam controls water flow by decreasing the water level in summer and increasing it in winter, which made it less favorable for snails to reproduce and survive. This, in turn, helped to decrease snail density, which is a crucial factor in the spread of schistosomiasis [32]. However, early flooding in the spring can have a negative effect on the survival and spawning rates of snails, which can impact snail density [127]. By adjusting the flood peaks, the Three Gorges Dam minimises the effect of early flooding on snail density, thus reducing the risk of schistosomiasis.

Sluice gates are structures made from either concrete or metal, which can control the amount of water flowing through rivers, canals, and other water bodies [128]. These gates can be raised or lowered to manage water levels, prevent flooding, and regulate water flow for hydroelectric power plants and irrigation systems [129]. In China, specially designed sluice gates have been utilized to prevent the spread of snails [130]. These gates have been installed in lakes and dams across China since 1998 to prevent snails from migrating or moving from the lake to the rivers or irrigation channels [131].

Japan explored the application of hot water and the use of flame throwers as snail control methods [107]. Though hot water and flame throwers may seem like effective solutions, they can have adverse environmental effects. The use of flame throwers, in particular, may lead to environmental damage, which is not a sustainable solution in the long run.

### 3.5. Recommendations for integrated control approaches

The key snail control practices for dams and irrigation schemes as well as their advantages and disadvantages as identified from this review are summarised in Table 2.

**Table 2:** Key snail control practices for dams and irrigation schemes.

Intervention types	Advantages	Disadvantages/Weaknesses
Water flow regulation	Reduced snail habitats and transmission	Increased salinity and waterlogging
Drainage projects	Reduced snail habitats and transmission	Expensive and time-consuming
Concrete lining of channels	Significant reduction in prevalence; Reduced snail habitats and transmission in irrigation canals; Reduced snail populations in rice field ditches	Expensive and requires ongoing maintenance and upkeep; Disrupts natural water flow, destroys habitats, reduces availability of nutrients for rice cultivation, increases the risk of flooding, and causes soil erosion
Mechanized irrigation systems	Decreased schistosomiasis in agricultural areas	Expensive to construct and maintain; May require significant infrastructure changes
Sluice gates	Decreased prevalence of schistosomiasis in agricultural and rural areas	Inadequate management can lead to environmental damage
Mechanical methods; Brushing and covering siphon boxes	Decreased prevalence by removing snails and larvae from water bodies; Decreased prevalence by reducing snail habitat	Labour-intensive and time consuming; brushing has a marginal effect on decreasing snail populations; may not be feasible on a large scale in areas with limited resources or access to technology
Water quality improvement and sanitation projects	Reduced exposure to contaminated water sources and snail habitats; break transmission cycle	Requires cross-sectoral collaboration and investment which can be difficult
Installation of fish ladders and introduction of natural predators	Reduced snail population and controlled schistosomiasis transmission in rivers and lakes; restored migration of river prawn	May disrupt the ecosystem and have unintended consequences
Introduction of crayfish and black carp	Reduced aquatic vegetation that supports snails as well as feeding on snails	May affect local biodiversity and fish
Saltwater intrusion and dredging	Altered the ecology of freshwater snails and reduced their abundance	May have negative effects on agriculture and fisheries
Electrification of wells	Decreased schistosomiasis in rural communities	Expensive to install and maintain; May require a reliable source of electricity
Changes in land use and agricultural practices	Reduced schistosomiasis transmission through improved water services and changes in agricultural practices	Conversion of natural habitats to agricultural or urban areas can lead to environmental degradation
Construction of bridges	Reduced exposure to contaminated water sources and snail habitats; break transmission cycle	Expensive to install

**Fig 6:**
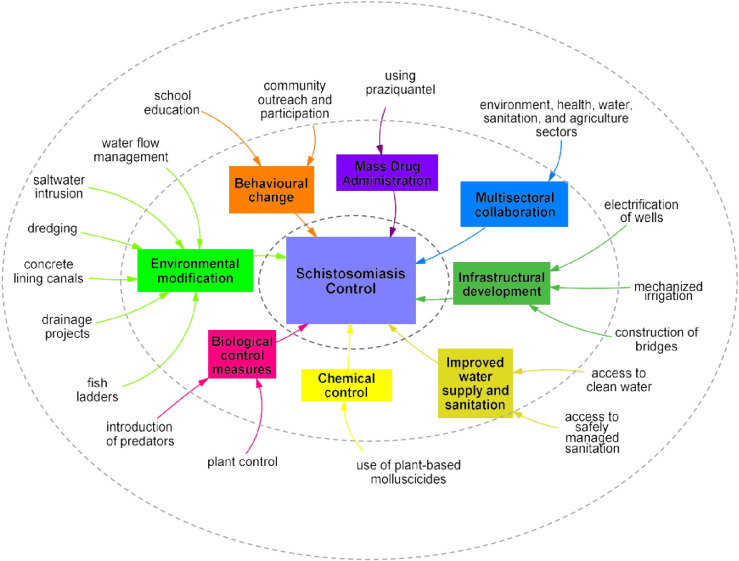
Integrated schistosomiasis control approaches recommended for areas with man-made water bodies (schistosomiasis control shown in the center requires overarching integrated control categories as shown in the inner ring/circle with corresponding appropriate intervention-types shown in the outer ring/circle).

A comprehensive approach to schistosomiasis control, where physical measures are combined with non-physical standard chemotherapy measures to ensure the long-term success of schistosomiasis control programmes, is an important strategy for areas of man-made water bodies. The results above highlight a variety of measures taken to prevent or overcome the challenges associated with managing both the snails and the disease. This range of interventions has, as common denominator, the integration of the necessary but clearly insufficient medical treatment based on praziquantel, with environmental interventions targeting the parasites when it is outside the human host [132,133]. Fig 6 highlights the overall integrated schistosomiasis control approaches recommended for areas where dams and irrigation schemes are built to improve water supply, provide energy, and enhance food security. We advocate for plant-based or other environmentally friendly molluscicides [134,135]. Community participation, planning and outreach programs should be developed to promote safe water practices and reduce the risk of schistosomiasis transmission [136]. This can include distributing educational materials, conducting workshops, and engaging with community leaders and stakeholders alongside infrastructure investments as part of integrated interventions [136,137]. Regarding integrated control approaches, multisectoral collaboration is crucial. Considering the role of environment, health, water, sanitation, energy and agriculture sectors in schistosomiasis control, multisectoral collaboration is highlighted as a recommended area for the elimination or control of schistosomiasis.

## 4. Discussion

Data are crucial in understanding the impact of water resources management on schistosomiasis. There were few detailed and recent studies carried out on the relationship between dams, irrigation schemes, schistosomiasis and engineering or biological snail control in many African countries, which has created a huge research gap. Schistosomiasis control requires the implementation of a combination of measures to address the various factors contributing to the transmission. The success of control measures depends on effective monitoring and evaluation of systems to assess their impact and effectiveness.

Ecological and climatic conditions are important factors. Understanding the occurrence of the snail intermediate host and its habitat is key to achieving effective snail control. This was clear in Morocco, where snail control was mainly conducted through gravity and focal mollusciciding [30]. The environmental and ecological studies on snails and their habitats were key in understanding the effectiveness of snail control measures in Japan [108]. Senegal’s Diama and Manantali dams had increased prevalence due to reduced salinity in the associated water bodies. In some instances, the climate conditions and changes in hydrology of a catchment area affected and reduced the prevalence of schistosomiasis. This was the case in Ouarzazate, Morocco, where continuous droughts disturbed the habitats of the snail intermediate hosts [52]. These examples clearly display how understanding the ecological, environmental and climatic condition plays a role in managing snail populations and especially because of climate change effects.

Increased water and sanitation access is critical in the control of schistosomiasis. Notably, countries such as Brazil, China, Japan, and Egypt implemented successful measures to improve access to safe water and sanitation, leading to a reduction in the transmission of the parasite. However, the success of such measures ultimately relies on political commitment and collaborative efforts across sectors to ensure the availability of safe water and sanitation facilities to affected communities. It is important to consider other effective infrastructural measures during the design of a dam or irrigation scheme such as providing the accompanying necessary water supply and sanitation infrastructure as part of the deliverables for water resources development.

Governance and Integrated Control Plans are important. Successful elimination of the disease requires investment and capacity building across multiple sectors. The countries which achieved success in controlling schistosomiasis followed a path of convergence which ensured relevant departments were involved in addressing schistosomiasis. This integrated approach of governance provided a conclusive policy framework and its implication to handling the spread of schistosomiasis and to eventually controlling it within the existing resources available. The coordination among health, environmental and agricultural bodies was apparent in China, Japan, Morocco and Egypt. Each country developed a schistosomiasis control program which reflected the need for social change, environmental adaptation, snail control and other measures such as development of WASH infrastructure. These cases provide evidence for the justification of an integrated approach for the purpose of containing schistosomiasis.

### 4.1. Limitations of the study

Many countries had inadequate reporting with few detailed recent studies carried out in many African countries. This limited the analysis to older information on the subject in some countries. Few articles that were relevant for the study were published in Arabic, French and Portuguese, meaning that some vital information on dams and schistosomiasis may have been missed or lost in translation by IEL.

There was no registration of our scoping review protocol in PROSPERO, and it was not reviewed and published in PROCEED, the global database of prospectively registered systematic reviews in the environmental sector. It was drafted using PRISMA-ScR principles and revised by the research team, one library staff and one member of faculty at Cranfield University only. It was not disseminated publicly to solicit additional feedback, which could have possibly impacted the search strategy and limited the results of the searches.

### 4.2. Conclusions

This review assessed the impact of dams and irrigation schemes on schistosomiasis prevalence in humans across Africa and assessed whether control measures implemented at various stages of the lifecycle of water management infrastructures have been able to curb the negative health outcomes of water resources development. The impact of dams and irrigation schemes when constructed with inadequate engineering and snail control measures resulted in significant increase in schistosomiasis. Concrete lining of canals, use of regulation/irrigation gates and construction of concrete banks were identified as the main engineering measures that contributed to successful snail control. Engineering measures worked better when combined with biological and other control measures including chemical control, improved water supply and sanitation, behavior change approaches and mass drug administration. Conversely, common failure factors in the control of schistosomiasis included poor dam design and irrigation channels, poor sanitation and hygiene, inadequate access to safe water, and insufficient monitoring and evaluation of the impact and effectiveness of control strategies. The failure factors must be addressed to improve the effectiveness of the control strategies and reduce the burden of schistosomiasis. Further considerations for research and water resources development implementation include the need for baseline data and inclusion of surveillance with subsequent M&E to adequately track changes over time. Additional evidence and research gap include developing environmentally friendly snail control plant molluscicides and identifying suitable natural predators. We provided key recommendations which could be adopted by the Continental Africa Water Investment Programme (AIP) as part of the Programme for Infrastructure Development in Africa Priority Action Plan. Recommendations on normative guidance and regulatory frameworks include detailed environmental and social impact assessments being strictly enforced, appropriate intervention design for infrastructure and environment, and improved cross sector coordination.

## Supporting information

S1 Text Search terms. (DOCX)

S2 Text Quality Appraisal Framework and Scoring Guidelines. (PDF)

S1 Table Details of dams and irrigation schemes included in the study. (PDF)

S1 File Scoping Review Protocol. (PDF)

S1 Checklist PRISMA Checklist. (PDF)

S1 Data Non Relevant Data Assessment. (DOCX)
