# Supplementary material for: Schistosomiasis and water resources development in Africa: A scoping review and multi-case evaluation of associated snail control
Source: PLoS Negl Trop Dis. 2025 Jun 12;19(6):e0013180. doi: 10.1371/journal.pntd.0013180 (PMC12193731; doi:10.1371/journal.pntd.0013180)
Supplement: S1 Text — (DOCX) [file pntd.0013180.s001.docx]

**Schistosomiasis and water resources development in Africa: A scoping review and multi-case evaluation of associated snail control**

Search Terms

| **Concept** | **Terms** |
| --- | --- |
| Schistosomiasis (SCH) | Schistosomiasis* or Schistosom or Bilharzia or Bilharz |
| Prevalence | endemic* or infection* or prevalence* |
| Water resources infrastructure | dam* or multipurpose dam or sand dam or concrete dam or irrigation* or irrigation project or irrigation scheme or irrigation canal or agriculture or barrage* or flow regulation* or drainage project* or channel* or concrete lining of channel or engineering control* |
| Snail control | snail control* or snail* or predator* or freshwater snail or control |
| Water supply and sanitation | water* or sanitation* or water supply or water treatment or piped water |
| Hydropower | hydro* or hydroelectric* |
| Continent | Africa* or Afric* |
| Country | Endemic countries in Africa |

The search terms were combined for example as follows:

Schistosomiasis* or Schistosom or Bilharzia or Bilharz AND endemic* or infection* or prevalence* AND dam* or multipurpose dam or sand dam or concrete dam or irrigation* or irrigation project or irrigation scheme or irrigation canal or agriculture or barrage* or flow regulation* or drainage project* or channel* or concrete lining of channel or engineering control* AND snail control* or snail* or predator* or freshwater snail or control or water* or sanitation* or water supply or water treatment or piped water or hydro* or hydroelectric* AND Africa* or Afric*. For Web of Science searches only, the keywords were then further combined with country names to identify any omitted country specific data from the countries, e.g., and “Egypt.”

For the different search engines:

| **Source** | **Search terms** |
| --- | --- |
| Web of Science | Schistosomiasis* or schistosoma or Bilharzia or Bilharz (Topic) and endemic* or infection* or prevalence* (Topic) and dam* or multipurpose dam or sand dam or concrete dam or irrigation* or irrigation project or irrigation scheme or irrigation canal or agriculture or barrage* or flow regulation* or drainage project* or channel* or concrete lining of channel or engineering control* (Topic) and snail control* or snail* or predator* or freshwater snail or control or water* or sanitation* or water supply or water treatment or piped water or hydro* or hydroelectric* (Topic) and Africa* or Afric* |
| PubMed Central | (schistosomiasis OR Schistosom* OR bilharzia OR bilharz*) AND (endemic OR infection OR prevalence) AND (dam OR multipurpose dam OR sand dam OR concrete dam OR irrigation OR irrigation project OR irrigation scheme OR irrigation canal OR barrage OR flow regulation OR drainage project OR channel OR concrete lining OR engineering control) AND (snail control OR snail OR predator OR freshwater snail) AND (water OR sanitation OR water supply OR water treatment OR pipe d water OR hydro* OR hydroelectric) AND (africa OR afric*) |
| Scopus | ( TITLE-ABS-KEY ( schistosomiasis OR schistosom* OR bilharzia OR bilharz* ) AND TITLE-ABS-KEY ( endemic* OR infection* OR infect* OR prevalence* ) AND TITLE-ABS-KEY ( dam* OR multipurpose OR sand OR concrete OR irrigation* OR scheme OR canal OR agriculture OR barrage* OR flow OR regulation* OR drainage OR drain* OR project* OR channel* OR concrete OR lining OR channel OR engineering OR control* ) AND TITLE-ABS-KEY ( snail OR control* OR predator* OR freshwater ) AND TITLE-ABS-KEY ( water* OR sanitation* OR supply OR treatment OR piped OR hydro* OR hydroelectric* OR hydropower ) AND TITLE-ABS-KEY ( africa OR afric* ) ) |
| WHO | Schistosomiasis, water, dams |
| FAO | schistosomiasis, snail control, dams, water resources management, prevalence |
| World Bank | schistosomiasis, snail control, dams, water resources management, prevalence |
