# Supplementary material for: Schistosomiasis and water resources development in Africa: A scoping review and multi-case evaluation of associated snail control
Source: PLoS Negl Trop Dis. 2025 Jun 12;19(6):e0013180. doi: 10.1371/journal.pntd.0013180 (PMC12193731; doi:10.1371/journal.pntd.0013180)
Supplement: S2 Text — (PDF) [file pntd.0013180.s002.pdf]

S1.2 Quality Appraisal Framework and Scoring Guidelines (1)

Topic	Criteria	Questions	Notes on scoring (out of 1)
Quality of Reporting	**1. Objectives**	**Were the objectives or aims of the study reported?**	0 = objectives not described, 0.5 = partly described, 1 = objectives and rationale thoroughly described.
	**2. Context**	**Was the context of the study described?**	0 = neither item below described, 0.5 = one item described, 1 = both items described.<br>• rationale for site selection<br>• broader social/historical/cultural/geographic context or implementation process
	**3. Study design**	**Was the type of study design mentioned?**	0 = study design not mentioned, 0.5 = study design not explicitly mentioned but elements are referred to, 1 = study design mentioned as retrospective, cross-sectional; longitudinal/panel; qualitative; ethnography, RCT, etc.
	**4. Sampling**	**Was the sampling method and sample described?**	0 = neither item below described, 0.5 = one item described, 1 = both items described.<br>• sampling strategy (e.g. random, purposive, etc.)<br>• sample size and description<br>If secondary data, should describe what portion of data were used and how data were collected (0.5 for each).
	**5. Data collection**	**Were data collection methods and procedures reported?**	0 = neither item below reported, 0.5 = one item reported, 1 = both items reported.<br>• type of primary data collected (e.g. surveys, interviews, water samples, observation)<br>• data collection instruments and process (collector, transcription/recording)<br>If secondary data: 1 = describe original data source, 0.5 = process of collecting original data not mentioned.
	**6. Analysis**	**Were analytical methods used in the study reported?**	0 = neither item below reported, 0.5 = one item reported, 1 = both items reported.<br>• analytical approach (e.g. content analysis or grounded theory; or estimator used, regression type)<br>• analytical process (e.g. coding transcripts, listening to recordings; or confidence interval, p-value, t-statistic)
Minimizing Risk of Bias	**7. Appropriatenes s of sampling**	**Was the sampling strategy appropriate to the study design?**	0 = sampling strategy not appropriate, 0.5 = sampling referred to, but appropriateness of strategy not described thoroughly or not discernable, 1 = sampling strategy appropriate.<br>• Quantitative studies should use representative sampling (some form of random sampling)<br>• Qualitative studies should theoretically use purposive, snowball, or convenience sampling<br>• Mixed methods studies may use a combination<br>If secondary data: 1 = original data source has representative sampling.
	**8. Appropriatenes s of data collection tools**	**Were appropriate data collection tools and measures used?**	0 = neither item below appropriate, 0.5 = one item appropriate, 1 = both items appropriate.<br>• e.g. surveys for quantitative, interviews/FDGs/observation for qualitative<br>• validated tools (for quantitative), triangulation of data/multiple sources (for qualitative)<br>If secondary data: 1 = original data source used appropriate/validated surveys. 0.5 = unclear or not verified.
	**9. Rigor and quality control in data collection**	**Was a rigorous data collection process adhered to?**	0 = neither item below practiced, 0.5 = one item practiced, 1 = both items practiced.<br>• pre-testing/piloting of data collection tools<br>• training and auditing of enumerators/data collectors (if NA, give 0)<br>If secondary data: 1 = original data source known to have rigorous processes (e.g. Census). 0.5 = authors describe rigor/QC to some extent or describe data cleaning efforts. 0 = not enough information present.

	**10. Rigor in data analysis**	**Were appropriate analytical methods used for the study design?**	0 = analytic methods inappropriate for study design, 0.5 = partial analytical rigor, 1 = appropriate methods.<br>e.g. appropriate statistical tests for quantitative data<br>e.g. appropriate qualitative data analysis procedures (e.g. coding, matrices)
	**11. External peer-review**	**Is there evidence of the study being subjected to external review?**	0 = no evidence of external review, 0.5 = internal review (e.g. dissertation), 1 = external peer review conducted.
**Conclusions**	**12. Interpretation**	**Is there a discussion and interpretation of the main findings?**	0 = no discussion, 0.5 = discussion incomplete, 1 = complete discussion and interpretation.
	**13. Limitations**	**Were study limitations described?**	0 = no limitations described, 0.5 = limitations incomplete, 1 = limitations thoroughly described.
	**14. Conclusions**	**Were stated conclusions within the scope of the study design?**	0 = conclusions not stated or beyond scope, 0.5 = partly beyond scope, 1 = conclusions within scope of study.

Studies were rated as high quality (score of ≥ 10 to 14), medium quality (score ≥ 5 and < 10), or low quality (score <5).

References:

1. Venkataramanan, V., Packman, A. I., Peters, D. R., Lopez, D., McCuskey, D. J., McDonald, R. I., Miller, W. M., & Young, S. L. (2019). A systematic review of the human health and social well-being outcomes of green infrastructure for stormwater and flood management. ***Journal of Environmental Management***, ***246***, 868–880. https://doi.org/10.1016/J.JENVMAN.2019.05.028
