# Supplementary material for: Schistosomiasis and water resources development in Africa: A scoping review and multi-case evaluation of associated snail control
Source: PLoS Negl Trop Dis. 2025 Jun 12;19(6):e0013180. doi: 10.1371/journal.pntd.0013180 (PMC12193731; doi:10.1371/journal.pntd.0013180)
Supplement: S1 Table — (PDF) [file pntd.0013180.s003.pdf]

Supplementary Table S1: Details of dams and irrigation schemes included in the study

Country	Dam and/or irrigation scheme Name	Purpose	Water Source	Year Completed /operational	Capacity	% Infection reported	% increase in Schistosomiasis due to dam	Engineering and biological snail control measures adopted during/before/after construction of the dam	Monitoring/ Evaluation	References
Algeria	El Hamiz River dam	N/A	Hamiz River	1869	16,300,000 m3	6%	No data	No data	No data	(1, 2, 3)
Angola	Capanda Dam	Hydro-electric power generation	Kwanza River	2004	4,795,000,000 m3	28%	No data	No data	NTD mapping committee	(4, 5, 6)
Benin	Okpara Dam	Water Supply	Oueme River	1970s	5,000,000 m3	20%	No data	No data	Mass treatment campaigns	(7, 8, 9, 10)
Botswana	Gaborone Dam	Water Supply	Notwane River	1963	141,100,000 m3	12%	No Data	No data	Nation Wide survey	(11)
Burkina Faso	Kompienga Dam	Hydro-electric power generation	Koulpélogo river	1985	2,000,000 m3	31%; 23% - 70% for *S. haematobium* and 0% - 69% for *S. mansoni*	No data	No data	National Program for Control with integrated M&E Component	(12, 13)
Burundi	Rwegura Dam	Irrigation	Rwegura River	2014	4,500,000m3	3%	No data	No data	No data	(14)
Cameroon	Lagdo dam	Multi-purpose	Benue River	1982	700 km2	61%	increase from 15% to 61% overall; *S. haernatobium* varied from 7-43% before; *S. mansoni* between 4 - 29% before; Over 100% increase recorded after	Concrete lined principal canals, concrete lined drains, regulating gates; biological fish control trial	No data	(2, 3, 15, 16, 17, 18, 19, 20, 21, 22, 23, 24)

Cameroon	Mape Dam	Multi-purpose	Mape River/Sanaga River Basin	1980s	4.2MW	1.7-81.1%	No data	Water treatment, removal of vegetation	No data	(21, 25, 26, 27)
Central African Republic	Boali Dam	Multi-purpose	Mbali River	1950s	23,000,000m3	10-15.8%	No data	No data	No data	(28)
Democratic Republic of Congo	Inga II Dam	Hydro-electric power generation	Congo River	1970s	1,424MW	31.40%	No data	No coordinated snail control efforts	No data	(29)
Cote d'Ivoire	Kossou Dam	Multi-purpose	Bandama River Basin	1972	174million m3	53%	marked increase in the prevalence of *S. haematobium* from 14% to 53%	Concrete spillaway on the left bank	No data	(21, 30, 31)
Cote d'Ivoire	Taabo Dam	Multi-purpose	Bandama River Basin	1978	1.8 billion m3	1-94%	prevalence of *S. haematobium* from 0% to 73%	Concrete lined principal canals, concrete lined drains, regulating gates	Monitored annually	(30, 32, 33, 34, 35, 36)
Djibouti	Ali Sabieh Dam	Irrigation	Ayboli River/ Awash River	1970	1.5 million m3	49-80%	No data	No data	No data	(37)
Egypt	Aswan High Dam	Hydro-electric power generation, Irrigation	Nile River	1970	43,000,000 m3	<1%	Increase from 21% to almost 100%	Crayfish introduction; Improved environmental and Irrigation practices were adopted to reduce snails; improved access to water supply and sanitation	Regular surveys; Significant reduction in prevalence to less than 1%	(38, 39, 40, 41, 42, 43, 44, 45, 46, 47, 48, 49, 50)
Eritrea	Mai Nefhi Dam	Irrigation/ Water supply	Anseba River	2016	160 million m3	21.5 - 40.2%;	No data	No data	No data	(51)

Ethiopia	Alwero Dam	irrigation	Alwero river	1985	74.6 milli on m3	72.3% reported by survey participants with claims of history of urogenital schistosomiasis infection; 6.4% collected snails infected	No data	No data	No data	(52)
Ethiopia	Awash dam/ Koka I,II, III.	Hydro-electric power generation, irrigation	Awash River/ valley	1960	180 km2	7.5% in 1968, 9% in 1972, 17% in 1975, 20% in 1980	No data	No data	No data	(53, 54, 55, 56)
Ghana	Akosombo (Volta) Dam	Hydro-electric power generation	Volta River	1965	7,900,00 0 m3	73%	70% and 75%	Biological and ecological control; manual and mechanical clearing of aquatic weeds that provide habitat and food for snails; surveys of snail distribution and prevalence of schistosomiasis in the Volta River basin	Regular monitoring	(4, 57, 58, 59, 60, 61, 62, 63, 64)
Kenya	Mwea Irrigation scheme	Irrigation	Mwea river basin	1957	6,000 hectares	60% children, 12% adults	No data	Clearing of the canals and streams of their thick vegetation, WASH interventions, bathing sites	No data	(24, 65)
Madagascar	Ankilivalo	Irrigation	Western Madagascar rice irrigation scheme (Ankilivalo)			69%	No data	No data	No data	(66)

Malawi	Nkula Dam	Hydro-electric power generation	Shire River	1978	24.8 million m3	40%	No data	Biological control measures	Regular monitoring	(67, 68, 69)
Mali	Selingui Dam	Hydro-electric power generation, irrigation	Sankarani river	1980	1.15 billion m3	*S. mansoni* 12.5% - 23.6%; Overall prevalence *S. haematobium* 85.9%; Prevalence of heavy infection *S. haematobium* 43.8%; Prevalence of heavy infection *S. haematobium* 43.8%;	*S. mansoni* increased from 0.9% to 12.5%; *S. haematobium* from 3.2% to 43.8%	Biological control measures	Regular Monitoring	(24, 70, 71, 72, 73)
Mauritania	Foum Gleita	Irrigation	Gorgol River	1986	500,000,000 m3; 4,000 ha (9,900 acres)	*S. haematobium* was 4.0%;	No data	No data	No data	(74)
Morrocco	Akka Oasis/Mouley Youssef	Irrigation	Akka riverbed/ Draa			Prevalence reduced from 6.8% in 1982 to 0.3% in 1997	Substantial evidence	Regular cleaning of syphon boxes, covering and brushing syphon boxes, Environmental management (community cleaning and clearing of aquatic vegetation in irrigation canals and impoundments, removing silt); building bridges,	Regular monitoring	(2, 24, 75, 76, 77, 78, 79, 80, 81)

Mozambique	Cahora Bassa Dam	Hydro-electric power generation	Zambezi River	1974	63,000,000 m3	50%	No Data	Control programme of keeping shorelines free of the Salvinia auriculata	Mapping, monitoring, and surveillance	(82, 83)
Niger	Malanvile Dam	Hydro-electric power generation, irrigation	Niger River	1983	130 million m3	15%; Decreased gradually in last 20 years from 27% to 15 %	No data	Biological control measures	Regular monitoring;	(84, 85)
Nigeria	Bakalori Dam	Hydro-electric power generation, irrigation	Sokoto River	1978	450 million m3	42.10%	No data	No data	No data	(86)
Nigeria	Erinle Dam	Water supply	Erinle River	old 1954; new 1989	old 5,300,000 m3; new 94,000,000 m3	43.0%-52.3%; *S. haematobium* 10% to 60%; *S. mansoni* 11.4% to 36.8%	No data	No data	No data	(87, 88, 89)
Nigeria	Malumfashi/ Tura Dam	Water supply and irrigation	River Tura	1983	6,000 m3	*S. mansoni* 4%	No data	No data	No data	(90)
Nigeria	Oyan River Dam	Hydro-electric power generation, Irrigation	Oyan River	1983	270,000,000 m3	50%	prevalence rates by presence of parasite ova were 90% in Abule-Titun, 88% in Apojola, 86% in Ibaro and 84% in Imala-Odo	Continuous discharge of water from the reservoir during the hot dry season reduced snail density by 90% and snail infection rate by 50%	No system for monitoring in place;	(91, 92, 93)
Nigeria	Tomas/Rimin Gado dam	Water supply and irrigation	Tomas River	1976	60.3 million m3	*S. haematobium* 26.6 - 36.8%	No data	No data	No data	(94)

Nigeria	Wasai Dam	Irrigation	Jakara and Getsi River	1976	559km2 and 1,659ha	*S. haematobium* 37%	No data	No data	No data	(95)
Nigeria	Warwade Dam	Irrigation and aquacultur e	Jigawa River	1977	300 million m3	*S. haematobium* 27.7% - 69.0%	No data	No data	No data	(96, 97)
Nigeria	Zobe Dam	Water supply and irrigation	Karaduwa River	1983	177 million m3	urinary schistosomiasi s 22.7 %	No data	No data	No data	(98)
Senegal	Diama dam	Prevent sal twater intrusion u pstream/irri gation	Senegal River	1986	250 million m3	43% to 99% for urogenital schistosomiasi s and between 2% and 95% for intestinal schistosomiasi s	up from nearly zero to up to 100%	Environmental management practices, restoring Macrobrachium vollenhovenii, the African river prawn, to the Senegal River	Regular monitoring	(99, 100, 101, 102, 103, 104, 105)
Senegal	Manantali dam	Hydro-electric power generation, irrigation	Bafing River	1988	11.3 Billion m3	25%-80%	up from about 10% to >80%	Environmental management practices, concrete linings: restoring Macrobrachium vollenhovenii, the African river prawn, to the Senegal River	Regular monitoring	(21, 99, 100, 101, 103, 106, 107)
Sierra Leone	Guma dam	Hydroelect ricity	Guma River	1966	220 million m3	*S. mansoni* 0% - 90%, *S. haematobium* 2.2% - 18.3%	No data	No data	No data	(108)
South Africa	Vaal Dam	Hydro-electric power generation, irrigation	Vaal River	1938	2511 million m3	11.07%	increased by 1.09%	Biological control measures, snail infestation significantly decreased in pools where white duck was introduced	Regular monitoring	(109, 110)
Sudan	Roseires Dam	Hydro-electric power generation, irrigation	Rahad River	2018	110 million m3	*S. mansoni* 14% - 70%, *S.haematobiu m* 1%	No data	No data	No data	(111, 112)

Tanzania	Baluchi Irrigation scheme	Irrigation	Mbarali River	1940	200 acres	*S. mansoni* 9.8%, *S. haematobium* 8.1%	No data	No data	No data	(113)
Tanzania	Ikowa Dam	Irrigation	No data	1959	1000 acres	*S. mansoni* absent. *S. haematobium* present	No data	No data	No data	(113)
Tanzania	Kalenga Irrigation scheme	Irrigation	Little Rusha River	1960	1000 acres	*S. mansoni* 0%, *S. haematobium* 6.7%	No data	No data	No data	(113)
Tanzania	Kalimawe Dam	Irrigation	No data	1958	1000 acres	*S. mansoni* ?, *S. haematobium* present	No data	No data	No data	(113)
Tanzania	Kilombera Irrigation scheme	Irrigation	Great Rusha River	1959	20000 acres	*S. mansoni* 7.7%, *S. haematobium* 22.5%	No data	No data	No data	(113)
Tanzania	Kisangara dam	Irrigation	Kisangara River	1963	116 ha	37%-86.3% urinary schistosomiasis	No data	Marisa cornuarietis, a well known ampullarid competitor/predator of Biomphalaria glabrata introduced	No data	(114, 115, 116)
Tanzania	Kisiwani Irrigation scheme	Irrigation	Kisiwani river	1961	1000 acres	*S. mansoni* 7.1%, *S. haematobium* 34.5%	No data	No data	No data	(113)
Tanzania	Kitivo Irrigation scheme	Irrigation	Umbo River	1960	1000 acres	*S. mansoni* 7.1%, *S. haematobium* 0%	No data	No data	No data	(113)
Tanzania	Mbarali Irrigation scheme	Irrigation	Mbarali River	1959	8000 acres	*S. mansoni* 9.8%, *S. haematobium* 8.1%	No data	No data	No data	(113)
Tanzania	Mangoini Irrigation scheme	Irrigation	Mianje Mungaa	1957	1000 acres	*S. mansoni* present. *S. haematobium* present	No data	No data	No data	(113)

Tanzania	Mtera Dam	Hydroelectricity	Great Ruaha River	1980	3,200 million m3	*S. haematobium* was 6.8% in Mtera Dam area and 38.7% in Uwandani Shehia	No data	No data	No data	(117)
Tanzania	Myumba ya Mungu Dam	Irrigation	Pangani River	1963	86000 acres	*S. mansoni* 95%, *S. haematobium* 50%	No data	No data	No data	(113)
Togo	Kara Dam	Hydro-electric power	Kara River	1960	15MW	51.5% - 79.6%	No data	No data	No data	(118)
Zimbabwe/ Zambia	Kariba Dam	Hydro-electric power generation, irrigation	Zambezi River	1959	94,000 million m3	*S. haematobium* 4.8% - 69%, *S. mansoni* 2.5% - 68%	No data	No data	Monitoring/Evaluation on Zimbabwe side	(119, 120)
Zimbabwe*	Mushandike dam	Irrigation Scheme	Mushandike River	1939	38.26 million m3	82% in 1987; *S. haematobium* 4.9%-12.7% and *S. mansoni* 0% - 6.5% in 1993	No data	Hydraulic structures such as sluice gates, weirs and outlets; Lined canals; regular drying of canals; water level fluctuation in night storage reservoirs; regular maintenance and routine cleaning; The in-field canal system included special features designed to flush snails (drop structures with stilling basins, special off takes); WASH.	Regular monitoring	(24, 119, 121, 122)

*Additionally, Hippo Valley sugar estates in Zimbabwe maintained a prevalence below 10% after a detailed control project in the 1970s placing greater emphasis on snail control: ducks as biological control agents and intensive water and sanitation component (Chimbari, 2012). The estates was not an original planned irrigation scheme but initiated irrigation to water its sugar plantations following market demand.

References

1. Hotez, P. J., Savioli, L., & Fenwick, A. (2012). Neglected tropical diseases of the Middle East and North Africa: review of their prevalence, distribution, and opportunities for control. PLoS neglected tropical diseases, 6(2), e1475. https://doi.org/10.1371/journal.pntd.0001475
2. Steinmann, P., Keiser, J., Bos, R., Tanner, M., & Utzinger, J. (2006). Schistosomiasis and water resources development: Systematic review, meta-analysis, and estimates of people at risk. Lancet Infect. Dis. 2006;6:411–425. doi: 10.1016/S1473-3099(06)70521-7
3. IAMAT (2015). World Schistosomiasis Risk Chart Geographical distribution of Schistosomiasis and principal snail vectors, 2015 edition https://www.iamat.org/assets/files/World%20Schistosomiasis%20Risk%20Chart_2015.pdf
4. Tchuem Tchuenté, LA., Rollinson, D., Stothard, J.R., Molyneux, D. (2017). Moving from control to elimination of schistosomiasis in sub-Saharan Africa: time to change and adapt strategies. Infect Dis Poverty 6, 42 (2017). https://doi.org/10.1186/s40249-017-0256-8
5. Allan, F., Sousa-Figueiredo, J. C., Emery, A. M., Paulo, R., Mirante, C., Sebastião, A., Brito, M., & Rollinson, D. (2017). Mapping freshwater snails in north-western Angola: distribution, identity and molecular diversity of medically important taxa. Parasites & vectors, 10(1), 460. https://doi.org/10.1186/s13071-017-2395-y
6. Mendes EP, Okhai H, Cristóvão RE, Almeida MC, Katondi N, Thompson R, et al. (2022) Mapping of schistosomiasis and soil-transmitted helminthiases across 15 provinces of Angola. PLoS Negl Trop Dis 16(6): e0010458. https://doi.org/10.1371/journal.pntd.0010458
7. Onzo-Aboki, A., Ibikounlé, M., Boko, P. M., Savassi, B. S., Doritchamou, J., Siko, E. J., Daré, A., Batcho, W., Massougbodji, A., Tougoue, J. J., & Kaboré, A. (2019). Human schistosomiasis in Benin: Countrywide evidence of Schistosoma haematobium predominance. Acta tropica, 191, 185–197. https://doi.org/10.1016/j.actatropica.2019.01.004
8. Ibikounlé, M., Mouahid, G., Mintsa Nguéma, R., Sakiti, N. G., Kindé-Gasard, D., Massougbodji, A., & Moné, H. (2012). Life-history traits indicate local adaptation of the schistosome parasite, Schistosoma mansoni, to its snail host, Biomphalaria pfeifferi. Experimental parasitology, 132(4), 501–507. https://doi.org/10.1016/j.exppara.2012.09.020
9. Boko, P. M., Ibikounle, M., Onzo-Aboki, A., Tougoue, J. J., Sissinto, Y., Batcho, W., Kinde-Gazard, D., & Kabore, A. (2016). Schistosomiasis and Soil Transmitted Helminths Distribution in Benin: A Baseline Prevalence Survey in 30 Districts. PloS one, 11(9), e0162798. https://doi.org/10.1371/journal.pone.0162798
10. Lai, Y. S., Biedermann, P., Ekpo, U. F., Garba, A., Mathieu, E., Midzi, N., Mwinzi, P., N'Goran, E. K., Raso, G., Assaré, R. K., Sacko, M., Schur, N., Talla, I., Tchuenté, L. A., Touré, S., Winkler, M. S., Utzinger, J., & Vounatsou, P. (2015). Spatial distribution of schistosomiasis and treatment needs in sub-Saharan Africa: a systematic review and geostatistical analysis. The Lancet. Infectious diseases, 15(8), 927–940. https://doi.org/10.1016/S1473-3099(15)00066-3
11. Gabaake, K. P., Phaladze, N. A., Lucero-Prisno Iii, D. E., & Thakadu, O. T. (2022). Assessment of awareness and knowledge of schistosomiasis among school-aged children (6-13 years) in the Okavango Delta, Botswana. Global health research and policy, 7(1), 36. https://doi.org/10.1186/s41256-022-00267-x
12. Koukounari, A., Touré, S., Donnelly, C. A., Ouedraogo, A., Yoda, B., Ky, C., Kaboré, M., Bosqué-Oliva, E., Basáñez, M. G., Fenwick, A., & Webster, J. P. (2011). Integrated monitoring and evaluation and environmental risk factors for urogenital schistosomiasis and active trachoma in Burkina Faso before preventative chemotherapy using sentinel sites. BMC infectious diseases, 11, 191. https://doi.org/10.1186/1471-2334-11-191
13. Poda, J. N., Traoré, A., & Sondo, B. K. (2004). L'endémie bilharzienne au Burkina Faso [Schistosomiasis endemic in Burkina Faso]. Bulletin de la Societe de pathologie exotique (1990), 97(1), 47–52.
14. Gryseels B. (1988). The morbidity of schistosomiasis mansoni in the Rusizi Plain (Burundi). Transactions of the Royal Society of Tropical Medicine and Hygiene, 82(4), 582–587. https://doi.org/10.1016/0035-9203(88)90519-6

15. Slootweg, R., Kooyman, M., de Koning, P., van Schooten, M. (1993). Water contact studies for the assessment of schistosomiasis infection risks in an irrigation scheme in Cameroon. Irrig Drainage Syst 7, 113–130 (1993). https://doi.org/10.1007/BF00880871
16. Slootweg, R., Malek, E.A. & McCullough, F.S. (1994). The biological control of snail intermediate hosts of schistosomiasis by fish. Rev Fish Biol Fisheries 4, 67–90 (1994). https://doi.org/10.1007/BF00043261
17. Massenet, D., Toukour, A., Kamwa Ngassam, R. I., Djao, R., Portal, J.-L., & Tchuenté, L.-A. T. (2011). Changes in the distribution of human schistosomiasis in Far North province, Cameroon, since 1986. Annals of Tropical Medicine & Parasitology, 105(4), 325–328. https://doi.org/10.1179/136485911X12987676649746
18. Green, A. E., Anchang-Kimbi, J. K., Wepnje, G. B., Ndassi, V. D., & Kimbi, H. K. (2021). Distribution and factors associated with urogenital schistosomiasis in the Tiko Health District, a semi-urban setting, South West Region, Cameroon. Infectious Diseases of Poverty, 10(1), 49. https://doi.org/10.1186/s40249-021-00827-2
19. Takougang, I., Meli, J., Fotso, S., Angwafo, F., Kamajeu, R., & Ndumbe, P. M. (2005). Some social determinants of urinary schistosomiasis in Northern Cameroon : implications for schistosomiasis control. African Journal of Health Sciences, 11(3). https://doi.org/10.4314/ajhs.v11i3.30788
20. Aula, O. P., McManus, D. P., Jones, M. K., & Gordon, C. A. (2021). Schistosomiasis with a Focus on Africa. Tropical Medicine and Infectious Disease, 6(3), 109. https://doi.org/10.3390/tropicalmed6030109
21. Sokolow, S. H., Jones, I. J., Jocque, M., La, D., Cords, O., Knight, A., Lund, A., Wood, C. L., Lafferty, K. D., Hoover, C. M., Collender, P. A., Remais, J. V., Lopez-Carr, D., Fisk, J., Kuris, A. M., & De Leo, G. A. (2017). Nearly 400 million people are at higher risk of schistosomiasis because dams block the migration of snail-eating river prawns. Philosophical Transactions of the Royal Society B: Biological Sciences, 372(1722), 20160127. https://doi.org/10.1098/rstb.2016.0127
22. Ratard, R. C., Kouemeni, L. E., Bessala, M. M., Ndamkou, C. N., Greer, G. J., Spilsbury, J., & Cline, B. L. (1990). Human schistosomiasis in Cameroon. I. Distribution of schistosomiasis. The American journal of tropical medicine and hygiene, 42(6), 561–572. https://doi.org/10.4269/ajtmh.1990.42.561
23. Brooker, S., Donnelly, C. A., & Guyatt, H. L. (2000). Estimating the number of helminthic infections in the Republic of Cameroon from data on infection prevalence in schoolchildren. Bulletin of the World Health Organization, 78(12), 1456–1465.
24. Boelee, E. & Madsen, H. (2006). Irrigation and schistosomiasis in Africa: ecological aspects. Colombo, Sri Lanka: International Water Management Institute (IWMI) 34p. (IWMI Research Report 99) [doi: 10.3910/2009.099]
25. Njunda, A. L., Ndzi, E. N., Assob, J. C. N., Kamga, H. F., & Kwenti, E. T. (2017). Prevalence and factors associated with urogenital schistosomiasis among primary school children in barrage, Magba sub-division of Cameroon. BMC public health, 17(1), 618. https://doi.org/10.1186/s12889-017-4539-6
26. Mewabo, A. P., Moyou, R. S., Kouemeni, L. E., Ngogang, J. Y., Kaptue, L., & Tambo, E. (2017). Assessing the prevalence of urogenital schistosomaisis and transmission risk factors amongst school-aged children around Mapé dam ecological suburbs in Malantouen district, Cameroon. Infectious diseases of poverty, 6(1), 40. https://doi.org/10.1186/s40249-017-0257-7
27. Mewamba, E. M., Tiofack, A. A. Z., Kamdem, C. N., Tchounkeu, E. Y., Tatang, R. J. A., Mengoue, L. E. T., Mbagnia, M. C. T., Njiokou, F., Casacuberta-Partal, M., Womeni, H. M., Simo, G., & Trypano GEN+ research group of the H3Africa consortium (2022). Fine-scale mapping of Schistosoma mansoni infections and infection intensities in sub-districts of Makenene in the Centre region of Cameroon. PLoS neglected tropical diseases, 16(10), e0010852.
28. Gras, C., Martet, G., Renoux, E., Lecamus, J. L., & Aubry, P. (1987). Une épidémie de bilharziose à schistosoma mansoni. 113 observations dans une collectivité militaire au retour d'Afrique centrale [An outbreak of Schistosoma mansoni bilharziasis. 113 cases in a military unit returning from Central Africa]. La Revue de medecine interne, 8(4), 379–382. https://doi.org/10.1016/s0248-8663(87)80009-7

29. Madinga, J., Linsuke, S., Mpabanzi, L., Meurs, L., Kanobana, K., Speybroeck, N., Lutumba, P., Polman, K. (2015). Schistosomiasis in the Democratic Republic of Congo: a literature review. Parasites Vectors 8, 601 (2015). https://doi.org/10.1186/s13071-015-1206-6
30. N'Goran, E. K., Diabate, S., Utzinger, J., & Sellin, B. (1997). Changes in human schistosomiasis levels after the construction of two large hydroelectric dams in central Côte d'Ivoire. Bulletin of the World Health Organization, 75(6), 541–545.
31. Diakité, N. R., Winkler, M. S., Coulibaly, J. T., Guindo-Coulibaly, N., Utzinger, J., & N'Goran, E. K. (2017). Dynamics of freshwater snails and Schistosoma infection prevalence in schoolchildren during the construction and operation of a multipurpose dam in central Côte d'Ivoire. Infectious diseases of poverty, 6(1), 93. https://doi.org/10.1186/s40249-017-0305-3
32. Angora, E. K., Boissier, J., Menan, H., Rey, O., Tuo, K., Touré, A. O., Coulibaly, J. T., Méité, A., Raso, G., N'Goran, E. K., Utzinger, J., & Balmer, O. (2019). Prevalence and Risk Factors for Schistosomiasis among Schoolchildren in two Settings of Côte d'Ivoire. Tropical medicine and infectious disease, 4(3), 110. https://doi.org/10.3390/tropicalmed4030110
33. M'Bra, R. K., Kone, B., Yapi, Y. G., Silué, K. D., Sy, I., Vienneau, D., Soro, N., Cissé, G., & Utzinger, J. (2018). Risk factors for schistosomiasis in an urban area in northern Côte d'Ivoire. Infectious diseases of poverty, 7(1), 47. https://doi.org/10.1186/s40249-018-0431-6
34. N'Goran, E. K., Utzinger, J., N'Guessan, A. N., Müller, I., Zamblé, K., Lohourignon, K. L., Traoré, M., Sosthène, B. A., Lengeler, C., & Tanner, M. (2001). Reinfection with Schistosoma haematobium following school-based chemotherapy with praziquantel in four highly endemic villages in Côte d'Ivoire. Tropical medicine & international health : TM & IH, 6(10), 817–825. https://doi.org/10.1046/j.1365-3156.2001.00785.x
35. Bassa, F. K., Eze, I. C., Assaré, R. K., Essé, C., Koné, S., Acka, F., Laubhouet-Koffi, V., Kouassi, D., Bonfoh, B., Utzinger, J., & N'Goran, E. K. (2022). Prevalence of Schistosoma mono- and co-infections with multiple common parasites and associated risk factors and morbidity profile among adults in the Taabo health and demographic surveillance system, South-Central Côte d'Ivoire. Infectious diseases of poverty, 11(1), 3. https://doi.org/10.1186/s40249-021-00925-1
36. Assaré, R. K., Tian-Bi, Y.-N. T., Yao, P. K., N’Guessan, N. A., Ouattara, M., Yapi, A., Coulibaly, J. T., Meïté, A., Hürlimann, E., Knopp, S., Utzinger, J., & N’Goran, E. K. (2016). Sustaining Control of Schistosomiasis Mansoni in Western Côte d’Ivoire: Results from a SCORE Study, One Year after Initial Praziquantel Administration. PLOS Neglected Tropical Diseases, 10(1), e0004329. https://doi.org/10.1371/journal.pntd.0004329
37. Koeck, J. L., Modica, C., Tual, F., Czarnecki, E., Fabre, R., Merle, C., Montfort, F., Jouvenin, N., & Cavallo, J. D. (1999). Découverte d'un foyer de bilharziose intestinale en République de Djibouti [Discovery of a focus of intestinal bilharziasis in te Republic of Djibouti]. Medecine tropicale : revue du Corps de sante colonial, 59(1), 35–38.
38. Barakat R. M. (2013). Epidemiology of Schistosomiasis in Egypt: Travel through Time: Review. Journal of advanced research, 4(5), 425–432. https://doi.org/10.1016/j.jare.2012.07.003
39. Abdel-Wahab M.F., El-Sahly A., Zakaria, Sohair, Strickland G.T., El-Kady N., Ahmed, Laila, (1979) CHANGING PATTERN OF SCHISTOSOMIASIS IN EGYPT 1935-79, The Lancet, Volume 314, Issue 8136, 1979, Pages 242-244, ISSN 0140-6736, https://doi.org/10.1016/S0140-6736(79)90249-6.
40. Abd-El Monsef, H., Smith, S. E., & Darwish, K. (2015). Impacts of the Aswan High Dam After 50 Years. Water Resources Management, 29(6), 1873–1885. https://doi.org/10.1007/s11269-015-0916-z
41. Malek, E. A. (1975). Effect of the Aswan High Dam on prevalence of schistosomiasis in Egypt. Tropical and Geographical Medicine, 27(4), 359–364.
42. Michelson, M. K., Azziz, F. A., Gamil, F. M., Wahid, A. A., Richards, F. O., Juranek, D. D., Habib, M. A., & Spencer, H. C. (1993). Recent Trends in the Prevalence and Distribution of Schistosomiasis in the Nile Delta Region. The American Journal of Tropical Medicine and Hygiene, 49(1), 76-87. https://doi.org/10.4269/ajtmh.1993.49.76
43. Richards, F. O., Wahid, A. A., Azziz, F. A., Gamil, F. M., Spencer, H. C., Michelson, M. K., Habib, M. A., & Juranek, D. D. (1993). Recent Trends in the Prevalence and Distribution of Schistosomiasis in

the Nile Delta Region. The American Journal of Tropical Medicine and Hygiene, 49(1), 76–87. https://doi.org/10.4269/ajtmh.1993.49.76
44. McNeeley, D. F., Cline, B. L., Hughes, J. M., El Alamy, M. A., Richards, F. O., El Hak, S., & Ruiz-Tiben, E. (1989). 1983 Nile Delta Schistosomiasis Survey: 48 Years after Scott. The American Journal of Tropical Medicine and Hygiene, 41(1), 56–62. https://doi.org/10.4269/ajtmh.1989.41.56
45. Haggag, A. A., Rabiee, A., Abd Elaziz, K. M., Gabrielli, A. F., Abdelhai, R., Hashish, A., Jabbour, J., & Ramzy, R. M. R. (2018). Elimination of schistosomiasis haematobia as a public health problem in five governorates in Upper Egypt. Acta Tropica, 188, 9–15. https://doi.org/10.1016/j.actatropica.2018.08.024
46. Biswas, A. K., & Tortajada, C. (2011). Impacts of the high Aswan Dam. In Impacts of large dams: A global assessment (pp. 379–395). Springer.
47. Ismail, N. M., Sedek, M. N, El-Said, K. M,, & Marai, A.M. A (2019). Effect of Water Bodies Lining on the Efficacy of Molluscicides Against Biomphalaria alexandrina Snails with Emphasis to their Integrated Control Measures. Egyptian Journal of Aquatic Biology and Fisheries, 23(5 (Special Issue)), 267–283. https://doi.org/10.21608/EJABF.2019.66051
48. Abou-El-Naga, I. F. (2013). Biomphalaria alexandrina in Egypt: Past, present and future. Journal of Biosciences, 38(3), 665–672. https://doi.org/10.1007/s12038-013-9329-4
49. Khater, H. F. (2017). Introductory Chapter: Back to the Future - Solutions for Parasitic Problems as Old as the Pyramids. In Natural Remedies in the Fight Against Parasites. InTech. https://doi.org/10.5772/67554
50. Abdel-Dayem, S., Abdel-Gawad, S., & Fahmy, H. (2007). Drainage in Egypt: a story of determination, continuity, and success. Irrigation and Drainage: The Journal of the International Commission on Irrigation and Drainage, 56(S1), S101–S111. https://doi.org/10.1002/ird.335
51. Chernet, A., Neumayr, A., Hatz, C., Kling, K., Sydow, V., Rentsch, K., Utzinger, J., Probst-Hensch, N., Marti, H., Nickel, B., & Labhardt, N. D. (2018). Spectrum of infectious diseases among newly arrived Eritrean refugees in Switzerland: a cross-sectional study. International journal of public health, 63(2), 233–239. https://doi.org/10.1007/s00038-017-1034-x
52. Deribew K, Erko B, Tiku Mereta S, Yewhalaw D, Mekonnen Z. (2022). Assessing Potential Intermediate Host Snails of Urogenital Schistosomiasis, Human Water Contact Behavior and Water Physico-chemical Characteristics in Alwero Dam Reservoir, Ethiopia. Environmental Health Insights. 2022;16. doi:10.1177/11786302221123576
53. Chala, B., & Torben, W. (2018). An Epidemiological Trend of Urogenital Schistosomiasis in Ethiopia. Frontiers in public health, 6, 60. https://doi.org/10.3389/fpubh.2018.00060
54. Kloos, H., & Lemma, A. (1977). Schistosomiasis in irrigation schemes in the Awash Valley, Ethiopia. The American journal of tropical medicine and hygiene, 26(5 Pt 1), 899–908. https://doi.org/10.4269/ajtmh.1977.26.899
55. Degarege, A., Mekonnen, Z., Levecke, B., Legesse, M., Negash, Y., Vercruysse, J., & Erko, B. (2015). Prevalence of Schistosoma haematobium Infection among School-Age Children in Afar Area, Northeastern Ethiopia. PloS one, 10(8), e0133142. https://doi.org/10.1371/journal.pone.0133142
56. Jemaneh, L., Tedla, S., & Birrie, H. (1994). The use of reagent strips for detection of urinary schistosomiasis infection in the middle Awash Valley, Ethiopia. East African medical journal, 71(10), 679–683.
57. Wen, S. T., & Chu, K. Y. (1984). Preliminary schistosomiasis survey in the lower Volta River below Akosombo Dam, Ghana. Annals of tropical medicine and parasitology, 78(2), 129–133. https://doi.org/10.1080/00034983.1984.11811786
58. Kabore A, Biritwum N-K, Downs PW, Soares Magalhaes RJ, Zhang Y, Ottesen EA (2013) Predictive vs. Empiric Assessment of Schistosomiasis: Implications for Treatment Projections in Ghana. PLoS Negl Trop Dis 7(3): e2051. https://doi.org/10.1371/journal.pntd.0002051
59. Hunter, J. M. (2003). Inherited burden of disease: agricultural dams and the persistence of bloody urine (Schistosomiasis hematobium) in the Upper East Region of Ghana, 1959–1997. Social Science & Medicine, 56(2), 219–234. https://doi.org/10.1016/s0277-9536(02)00021-7

60. Hotez, P. J., Biritwum, N.-K., Fenwick, A., Molyneux, D. H., & Sachs, J. D. (2019). Ghana: Accelerating neglected tropical disease control in a setting of economic development. PLOS Neglected Tropical Diseases, 13(1), e0007005. https://doi.org/10.1371/journal.pntd.0007005
61. Zakhary K., (1997). Factors affecting the prevalence of schistosomiasis in the Volta Region of Ghana. McGill Journal of Medicine. 1997;3(2):93–101. [Internet]. 2020 Dec. https://doi.org/10.26443/mjm.v3i2.583
62. Nyekodzi, G., Lawson, E. T., & Gordon, C. (2018). Evaluating the impacts of dredging and saline water intrusion on rural livelihoods in the Volta Estuary. International Journal of River Basin Management, 16(1), 93–105. https://doi.org/10.1080/15715124.2017.1372445
63. Kranjac-Berisavljevic, G., & Abagale, F. K. (2004). Siltation Of Dams And Reservoirs And Its Effect On Agricultural Production In Northern Ghana: A Case Study Of Chabchab Irrigation Project, Savelugu-Nanton District. http://www.udsspace.uds.edu.gh/handle/123456789/2169?mode=full [Accessed 07/03/2023]
64. Schur, N., Hürlimann, E., Garba, A., Traoré, M. S., Ndir, O., Ratard, R. C., Tchuem Tchuenté, L.-A., Kristensen, T. K., Utzinger, J., & Vounatsou, P. (2011). Geostatistical Model-Based Estimates of Schistosomiasis Prevalence among Individuals Aged ≤20 Years in West Africa. PLoS Neglected Tropical Diseases, 5(6), e1194. https://doi.org/10.1371/journal.pntd.0001194
65. Masaku, J., Madigu, N., Okoyo, C., & Njenga, S. M. (2015). Current status of Schistosoma mansoni and the factors associated with infection two years following mass drug administration programme among primary school children in Mwea irrigation scheme: A cross-sectional study. BMC public health, 15, 739. https://doi.org/10.1186/s12889-015-1991-z
66. Howarth, S. E., Wilson, J. M., Ranaivoson, E., Crook, S. E., Denning, A. M., & Hutchings, M. S. (1988). Worms, wells and water in western Madagascar. The Journal of tropical medicine and hygiene, 91(5), 255–264.
67. Jones, C. M., Wilson, A. L., Stanton, M. C., Stothard, J. R., Guglielmo, F., Chirombo, J., Mafuleka, L., Oronje, R., & Mzilahowa, T. (2023). Integrating vector control within an emerging agricultural system in a region of climate vulnerability in southern Malawi: A focus on malaria, schistosomiasis, and arboviral diseases. Current research in parasitology & vector-borne diseases, 4, 100133. https://doi.org/10.1016/j.crpvbd.2023.100133
68. Makaula, P., Sadalaki, J. R., Muula, A. S., Kayuni, S., Jemu, S., & Bloch, P. (2014). Schistosomiasis in Malawi: a systematic review. Parasites & vectors, 7, 570. https://doi.org/10.1186/s13071-014-0570-y
69. Poole, H., Terlouw, D. J., Naunje, A., Mzembe, K., Stanton, M., Betson, M., Lalloo, D. G., & Stothard, J. R. (2014). Schistosomiasis in pre-school-age children and their mothers in Chikhwawa district, Malawi with notes on characterization of schistosomes and snails. Parasites & vectors, 7, 153. https://doi.org/10.1186/1756-3305-7-153
70. Sacko, M., Magnussen, P., Keita, A. D., Traoré, M. S., Landouré, A., Doucouré, A., Madsen, H., & Vennervald, B. J. (2011). Impact of Schistosoma haematobium infection on urinary tract pathology, nutritional status and anaemia in school-aged children in two different endemic areas of the Niger River Basin, Mali. Acta tropica, 120 Suppl 1, S142–S150. https://doi.org/10.1016/j.actatropica.2010.12.009
71. Clements, A. C., Bosqué-Oliva, E., Sacko, M., Landouré, A., Dembélé, R., Traoré, M., Coulibaly, G., Gabrielli, A. F., Fenwick, A., & Brooker, S. (2009). A comparative study of the spatial distribution of schistosomiasis in Mali in 1984-1989 and 2004-2006. PLoS neglected tropical diseases, 3(5), e431. https://doi.org/10.1371/journal.pntd.0000431
72. Traoré M. (1989). Schistosomiasis in the Sélingué dam area: the integrated approach. Tropical medicine and parasitology : official organ of Deutsche Tropenmedizinische Gesellschaft and of Deutsche Gesellschaft fur Technische Zusammenarbeit (GTZ), 40(2), 228–231.
73. Traore, M., Traore, H. A., Kardorff, R., Diarra, A., Landoure, A., Vester, U., Doehring, E., & Bradley, D. J. (1998). The public health significance of urinary schistosomiasis as a cause of morbidity in two districts in Mali. The American journal of tropical medicine and hygiene, 59(3), 407–413. https://doi.org/10.4269/ajtmh.1998.59.407
74. Gbalégba, N. G. C., Silué, K. D., Ba, O., Ba, H., Tian-Bi, N. T. Y., Yapi, G. Y., Kaba, A., Koné, B., Utzinger, J., & Koudou, B. G. (2017). Prevalence and seasonal transmission of Schistosoma

haematobium infection among school-aged children in Kaedi town, southern Mauritania. Parasites & vectors, 10(1), 353. https://doi.org/10.1186/s13071-017-2284-4
75. Balahbib, A., Amarir, F., Bouhout, S., Rhajaoui, M., Adlaoui, E., & Sadak, A. (2020). Review of the Urinary Schistosomiasis Control in Morocco (1960-2018). Interdisciplinary perspectives on infectious diseases, 2020, 3868970. https://doi.org/10.1155/2020/3868970
76. Laamrani, H., Khallaayoune, K., Boelee, E., Laghroubi, M.M., Madsen, H. and Gryseels, B. (2000a), Evaluation of environmental methods to control snails in an irrigation system in Central Morocco. Tropical Medicine & International Health, 5: 545-552. https://doi.org/10.1046/j.1365-3156.2000.00606.x
77. Boelee, E. & Laamrani, H. (2004). Environmental control of schistosomiasis through community participation in a Moroccan oasis. Tropical medicine & international health : TM & IH, 9(9), 997–1004. https://doi.org/10.1111/j.1365-3156.2004.01301.x
78. Laamrani, H., Mahjour, J., Madsen, H., Khallaayoune, K., & Gryseels, B. (2000b). Schistosoma haematobium in Morocco: moving from control to elimination. Parasitology today, 16(6), 257–260. ISSN 0169-4758. https://doi.org/10.1016/s0169-4758(00)01665-3
79. Amarir, F., El Mansouri, B., Fellah, H., Sebti, F., Mohammed, L., Handali, S., Wilkins, P., El Idrissi, A. L., Sadak, A., & Rhajaoui, M. (2011). National serologic survey of Haematobium schistosomiasis in Morocco: evidence for elimination. The American journal of tropical medicine and hygiene, 84(1), 15–19. https://doi.org/10.4269/ajtmh.2011.10-0378
80. Barkia, H., Barkia, A., Yacoubi, R., Alemad, A., El Kharim, K., & Belghyti, D. (2014). Contribution of Mobile Teams to Efforts to Eliminate Schistosomiasis at Schistosoma haematobium in Morocco-Narrative Review Article. Iranian journal of public health, 43(9), 1167–1175.
81. Khallaayoune, K., Madsen, H., & Laamrani, H. (1998b). Evaluation of three methods to control Bulinus truncatus, the intermediate host of Schistosoma haematobium in an irrigation scheme, Tessaout-Amont, Morocco, Acta Tropica, Volume 69, Issue 1, 1998, Pages 51-63, ISSN 0001-706X. https://doi.org/10.1016/S0001-706X(97)00119-8.
82. Phillips, A. E., Gazzinelli-Guimaraes, P. H., Aurelio, H. O., Ferro, J., Nala, R., Clements, M., King, C. H., Fenwick, A., Fleming, F. M., & Dhanani, N. (2017). Assessing the benefits of five years of different approaches to treatment of urogenital schistosomiasis: A SCORE project in Northern Mozambique. PLoS neglected tropical diseases, 11(12), e0006061. https://doi.org/10.1371/journal.pntd.0006061
83. Isaacman, A. (2021). Cahora Bassa Dam & the Delusion of Development. Daedalus 2021; 150 (4): 103–123. doi: https://doi.org/10.1162/daed_a_01875
84. Labbo, R., Garba, A., Louboutin-Croc, J. P., Ernould, J. C., Sellin, B., Chippaux, J. P., & Stothard, J. R. (2003). The spread of Biomphalaria pfeifferi in the Niger River valley, Niger. Annals of tropical medicine and parasitology, 97(2), 209–212. https://doi.org/10.1179/000349803235001507
85. Garba, A., Labbo, R., Tohon, Z., Sidiki, A., & Djibrilla, A. (2004). Emergence of Schistosoma mansoni in the Niger River valley, Niger. Transactions of the Royal Society of Tropical Medicine and Hygiene, 98(5), 296–298. https://doi.org/10.1016/S0035-9203(03)00070-1
86. Umar, A. S., & Parakoyi, D. B. (2005). The Prevalence and Intensity of Urinary Schistoso-miasis Among School Children Living along the Bakalori Dam, Nigeria. The Nigerian postgraduate medical journal, 12(3), 168–172.
87. Oladejo, S. O., & Ofoezie, I. E. (2006). Unabated schistosomiasis transmission in Erinle River Dam, Osun State, Nigeria: evidence of neglect of environmental effects of development projects. Tropical medicine & international health : TM & IH, 11(6), 843–850. https://doi.org/10.1111/j.1365-3156.2006.01628.x
88. Adewunmi, C. O., Gebremedhin, G., Becker, W., Olurunmola, F. O., Dörfler, G., & Adewunmi, T. A. (1993). Schistosomiasis and intestinal parasites in rural villages in southwest Nigeria: an indication for expanded programme on drug distribution and integrated control programme in Nigeria. Tropical medicine and parasitology : official organ of Deutsche Tropenmedizinische Gesellschaft and of Deutsche Gesellschaft fur Technische Zusammenarbeit (GTZ), 44(3), 177–180.
89. Ugbomoiko, U. S., & Ofoezie, I. E. (2007). Multiple infection diagnosis of intestinal helminthiasis in the assessment of health and environmental effect of development projects in Nigeria. Journal of Helminthology, 81(3), 227–231. doi:10.1017/S0022149X07685381

90. Pugh, R. N. H., Burrows, J. W., & Bradley, A. K. (1981). Malumfashi Endemic Diseases Research Project, XVI: The findings of a survey for schistosomiasis mansoni. Hookworm, giardiasis and nutritional status. Annals of Tropical Medicine & Parasitology, 75(3), 281–292. https://doi.org/10.1080/00034983.1981.11687442
91. Bayegun, A. A., Omitola, O. O., Umunnakwe, C. U., Akande, F. A., Akinwale, O. P., Mogaji, H. O., Ademolu, K. O., Gyang, V. P., Odoemene, S. N., Stothard, J. R., & Ekpo, U. F. (2023). Morphometric analysis of schistosome eggs recovered from human urines in communities along the shoreline of Oyan River Dam in Ogun State, Nigeria. Journal of helminthology, 96, e89. https://doi.org/10.1017/S0022149X22000815
92. Akinwale, O. P., Oliveira, G. C., Ajayi, M. B., Akande, D. O., Oyebadejo, S., & Okereke, K. C. (2008). Squamous cell abnormalities in exfoliated cells from the urine of Schistosoma haematobium-infected adults in a rural fishing community in Nigeria. World health & population, 10(1), 18–22. https://doi.org/10.12927/whp.2008.19581
93. Ofoezie, I. E., Imevbore, A. M., Balogun, M. O., Ogunkoya, O. O., & Asaolu, S. O. (1991). A study of an outbreak of schistosomiasis in two resettlement villages near Abeokuta, Ogun State, Nigeria. Journal of helminthology, 65(2), 95–102. https://doi.org/10.1017/s0022149x00010531
94. Betterton, C., Ndifon, G. T., Bassey, S. E., Tan, R. M., & Oyeyi, T. (1988). Schistosomiasis in Kano State, Nigeria. I. Human infections near dam sites and the distribution and habitat preferences of potential snail intermediate hosts. Annals of tropical medicine and parasitology, 82(6), 561–570.
95. Enabulele, E. E., Platt, R. N., Adeyemi, E., Agbosua, E., Aisien, M. S. O., Ajakaye, O. G., Ali, M. U., Amaechi, E. C., Atalabi, T. E., Auta, T., Awosolu, O. B., Dagona, A. G., Edo-Taiwo, O., Ejikeugwu, C. E. P., Igbeneghu, C., Njom, V. S., Orji, M. N., Oyinloye, F. O. P., Ozemoka, H. J., Ugah, U. I., Anderson, T. J. C. (2021). Urogenital schistosomiasis in Nigeria post receipt of the largest single praziquantel donation in Africa. Acta tropica, 219, 105916. https://doi.org/10.1016/j.actatropica.2021.105916
96. Muhammed, H., Balogun, J. B., Dogara, M. M., Adewale, B., Ibrahim, A. A., Okolugbo, C. B., & Jackson, G. (2023). Co-infection of urogenital schistosomiasis and malaria and its association with anaemia and malnutrition amongst schoolchildren in Dutse, Nigeria. South African Journal of Science, 119(7/8). https://doi.org/10.17159/sajs.2023/13846
97. Balogun, J. B., Adewale, B., Balogun, S. U., Lawan, A., Haladu, I. S., Dogara, M. M., Aminu, A. U., Caffrey, C. R., De Koning, H. P., Watanabe, Y., & Balogun, E. O. (2022). Prevalence and Associated Risk Factors of Urinary Schistosomiasis among Primary School Pupils in the Jidawa and Zobiya Communities of Jigawa State, Nigeria. Annals of global health, 88(1), 71. https://doi.org/10.5334/aogh.3704
98. Atalabi, T. E., Lawal, U., & Ipinlaye, S. J. (2016). Prevalence and intensity of genito-urinary schistosomiasis and associated risk factors among junior high school students in two local government areas around Zobe Dam in Katsina State, Nigeria. Parasites & vectors, 9(1), 388. https://doi.org/10.1186/s13071-016-1672-5
99. Southgate, V. R. (1997). Schistosomiasis in the Senegal River Basin: before and after the construction of the dams at Diama, Senegal and Manantali, Mali and future prospects. Journal of Helminthology, 71(2), 125–132.
100. Talla, I., Kongs, A., Verlé, P., Belot, J., Sarr, S., & Coll, A. M. (1990). Outbreak of intestinal schistosomiasis in the Senegal River Basin. Annales de la Societe belge de medecine tropicale, 70(3), 173–180.
101. Wood, C. L., Sokolow, S. H., Jones, I. J., Chamberlin, A. J., Lafferty, K. D., Kuris, A. M., Jocque, M., Hopkins, S., Adams, G., Buck, J. C., Lund, A. J., Garcia-Vedrenne, A. E., Fiorenza, E., Rohr, J. R., Allan, F., Webster, B., Rabone, M., Webster, J. P., Bandagny, L., Ndione, R., … De Leo, G. A. (2019). Precision mapping of snail habitat provides a powerful indicator of human schistosomiasis transmission. Proceedings of the National Academy of Sciences of the United States of America, 116(46), 23182–23191. https://doi.org/10.1073/pnas.1903698116
102. Sow, S., de Vlas, S. J., Engels, D., & Gryseels, B. (2002). Water-related disease patterns before and after the construction of the Diama dam in northern Senegal. Annals of tropical medicine and parasitology, 96(6), 575–586. https://doi.org/10.1179/000349802125001636

103. Sow, S., de Vlas, S.J., Stelma, F., Vereecken, K., Gryseels, B., & Polman, K. (2011). The contribution of water contact behavior to the high Schistosoma mansoni Infection rates observed in the Senegal River Basin. BMC Infect Dis 11, 198 (2011). https://doi.org/10.1186/1471-2334-11-198
104. Savaya Alkalay, A., Rosen, O., Sokolow, S. H., Faye, Y. P. W., Faye, D. S., Aflalo, E. D., Jouanard, N., Zilberg, D., Huttinger, E., & Sagi, A. (2014). The Prawn Macrobrachium vollenhovenii in the Senegal River Basin: Towards Sustainable Restocking of All-Male Populations for Biological Control of Schistosomiasis. PLoS Neglected Tropical Diseases, 8(8), e3060. https://doi.org/10.1371/journal.pntd.0003060
105. Rohr, J. R., Sack, A., Bakhoum, S., Barrett, C. B., Lopez-Carr, D., Chamberlin, A. J., Civitello, D. J., Diatta, C., Doruska, M. J., De Leo, G. A., Haggerty, C. J. E., Jones, I. J., Jouanard, N., Lund, A. J., Ly, A. T., Ndione, R. A., Remais, J. V., Riveau, G., Schacht, A. M., Seck, M., … Wolfe, C. (2023). A planetary health innovation for disease, food and water challenges in Africa. Nature, 619(7971), 782–787. https://doi.org/10.1038/s41586-023-06313-z
106. Picquet, M., Ernould, J. C., Vercruysse, J., Southgate, V. R., Mbaye, A., Sambou, B., Niang, M., & Rollinson, D. (1996). The epidemiology of human schistosomiasis in the Senegal river basin. Transactions of the Royal Society of Tropical Medicine and Hygiene, 90(4), 340–346.
107. Shaikh, N., Rahman-Shepherd, A., & Dar, O. (2018). Schistosomiasis in the Senegal River basin. The Lancet Planetary Health, 2, S27. https://doi.org/10.1016/S2542-5196(18)30112-8
108. Hodges, M., Dada, N., Wamsley, A., Paye, J., Nyorkor, E., Sonnie, M., Barnish, G., Bockarie, M., & Zhang, Y. (2011). Improved mapping strategy to better inform policy on the control of schistosomiasis and soil-transmitted helminthiasis in Sierra Leone. Parasites & vectors, 4, 97. https://doi.org/10.1186/1756-3305-4-97
109. Pretorius, S. J., Joubert, P. H., & de Kock, K. N. (1989). A review of the schistosomiasis risk in South African dams. Water SA Vol. 15. No.2. April 1989 ISSN 0378-4738 https://journals.co.za/doi/pdf/10.10520/AJA03784738_1206 https://www.wrc.org.za/wp-content/uploads/mdocs/WaterSA_1989_02_530.PDF
110. de Kock, K. N. & Wolmarans, C. T. (2005). Distribution and habitats of the Bulinus africanus species group, snail intermediate hosts of Schistosoma haematobium and S. mattheei in South Africa. Water SA Vol. 31 No. 1 (2005) 117-125. DOI: 10.4314/wsa.v31i1.5128
111. Tameim, O., Zakaria, Z. B., Hussein, H., el Gaddal, A. A., Jobin, W. R. (1985). Control of schistosomiasis in the new Rahad Irrigation Scheme of Central Sudan. The Journal of Tropical Medicine and Hygiene. 1985 Apr;88(2):115-124. PMID: 4032520.
112. Meyer-Lassen, J., Daffalla, A. A. & Madsen, H. (1994). Evaluation of focal mollusciciding in the Rahad Irrigation Scheme, Sudan, Acta Tropica, Volume 58, Issues 3–4, 1994, Pages 229-241, ISSN 0001-706X, https://doi.org/10.1016/0001-706X(94)90017-5.
113. Sturrock, R. F. (1965). The development of irrigation and its influence on the transmission of bilharziasis in Tanganyika. Bulletin of the World Health Organization, 32(2), 225–236.
114. Madsen H. (1983). Distribution of Helisoma duryi, an introduced competitor of intermediate hosts of schistosomiasis, in an irrigation scheme in northern Tanzania. Acta tropica, 40(3), 297–306.
115. Nguma, J. F., McCullough, F. S., & Masha, E. (1982). Elimination of Biomphalaria pfeifferi, Bulinus tropicus and Lymnaea natalensis by the ampullarid snail, Marisa cornuarietis, in a man-made dam in northern Tanzania. Acta tropica, 39(1), 85–90.
116. Poggensee, G., Krantz, I., Nordin, P., Mtweve, S., Ahlberg, B., Mosha, G., & Freudenthal, S. (2005). A six-year follow-up of schoolchildren for urinary and intestinal schistosomiasis and soil-transmitted helminthiasis in Northern Tanzania. Acta tropica, 93(2), 131–140. https://doi.org/10.1016/j.actatropica.2004.10.003
117. Ngasala, B., Juma, H., & Mwaiswelo, R. O. (2020). The usefulness of indirect diagnostic tests for Schistosoma haematobium infection after repeated rounds of mass treatment with praziquantel in Mpwapwa and Chakechake districts in Tanzania. International journal of infectious diseases : IJID : official publication of the International Society for Infectious Diseases, 90, 132–137. https://doi.org/10.1016/j.ijid.2019.10.031
118. Lapierre, J., Tourte-Schaefer, C., Dupouy-Camet, J., Heyer, F., & Faurant, C. (1988). Etude épidémiologique du foyer de bilharziose à Schistosoma mansonide Kara (Nord Togo) [An

epidemiologic study of a focus of Schistosoma mansoni bilharziasis in Kara (North Togo)]. Bulletin de la Societe de pathologie exotique et de ses filiales, 81(5), 861–868.

119. Chimbari M. J. (2012). Enhancing schistosomiasis control strategy for zimbabwe: building on past experiences. Journal of Parasitology Research, 2012, 353768. https://doi.org/10.1155/2012/353768

120. Mungomba, L. M., Chandiwana, S. K., & Madesen, H. (1993). Schistosomiasis around Siavonga, on the shores of Lake Kariba, Zambia. Annals of tropical medicine and parasitology, 87(4), 365–371. https://doi.org/10.1080/00034983.1993.11812780

121. Chandiwana, S. K., Taylor, P., Chimbari, M., Ndhlovu, P., Makura, O., Bradley, M., & Gondo, P. (1988). Control of schistosomiasis transmission in newly established smallholder irrigation schemes. Transactions of the Royal Society of Tropical Medicine and Hygiene, 82(6), 874–880. https://doi.org/10.1016/0035-9203(88)90024-7

122. Chimbari, M., Ndlela, B., Nyati, Z., Thomson, A., Chandiwana, S. K., & Bolton, P. (1992). Bilharzia in a small irrigation community: an assessment of water and toilet usage. The Central African journal of medicine, 38(12), 451–458.
