# Supplementary material for: Schistosomiasis and water resources development in Africa: A scoping review and multi-case evaluation of associated snail control
Source: PLoS Negl Trop Dis. 2025 Jun 12;19(6):e0013180. doi: 10.1371/journal.pntd.0013180 (PMC12193731; doi:10.1371/journal.pntd.0013180)
Supplement: S1 File — (PDF) [file pntd.0013180.s004.pdf]

Working Title: Schistosomiasis and water resources development in Africa: A scoping review and multi-case evaluation of associated snail control

Study protocol for Scoping Review of Schistosomiasis and water resources development in Africa and a multi-case evaluation of associated snail control

Objective

To examine the impacts of infrastructural man-made water bodies such as dams and irrigation schemes on schistosomiasis transmission and further reflect on the successes and failures of implementation of engineering techniques and biological snail control measures.

Authors

May N Sule[a*], Ibrahim El Lahham[a], Mutinta N Munkombwe[a], Patricia Nasike[a], Anouk Gouvras[b], David Rollinson[b], Rashid Mbaziira[c], Comfort Kanshio[c], Giulio A De Leo[d]

[a] Centre for Water, Environment and Development, Cranfield University, College Road, Cranfield, MK43 0AL, UK
[b]Global Schistosomiasis Alliance, London, UK
[c]African Ministers' Council on Water (AMCOW), 11 TY Danjuma Street, Asokoro Abuja, Nigeria
[d] Department of Oceans and Woods Institute for the Environment, Hopkins Marine Station, Stanford University, Pacific Grove, CA, USA

Reporting

We adhered to the Preferred Reporting Items for Systematic Reviews and Meta-Analyses - Scoping Review statement, PRISMA-ScR (1) and the JBI Manual for Evidence Synthesis (2).

Search methods

We searched Web of Science, Pubmed and Scopus for this review. We also searched the online databases of WHO, the Food and Agriculture Organization of the United Nations' (FAO) Global Information System on Water and Agriculture AQUASTAT. Books and other grey literature such as dissertations were also considered. We further scanned the bibliographies of several systematic reviews including Steinmann et al. (3) and Sokolow et al. (4). We considered papers published at any time without any date exclusions, and in English language. We also considered articles in French, Arabic and Portuguese language. We searched articles cited in another paper that appeared relevant and considered such for inclusion to be reviewed where suitable.

Search terms

Our searches included each of the following schistosomiasis and water resources development terms in conjunction with associated snail control related terms.

Web of Science:

Schistosomiasis* or Schistosom or Bilharzia or Bilharz AND endemic* or infection* or prevalence* AND dam* or multipurpose dam or sand dam or concrete dam or irrigation* or irrigation project or irrigation scheme or irrigation canal or agriculture or barrage* or flow regulation* or drainage project* or channel* or concrete lining of channel or engineering control* AND snail control* or snail* or predator* or freshwater snail or control or water* or

sanitation* or water supply or water treatment or piped water or hydro* or hydroelectric* AND Africa* or Afric*. The keywords were then further combined with country names to identify any omitted country specific data, e.g., and “Egypt.”

Web of Science: 1091 results
Pubmed: 622 results
Scopus: 836 results
WHO: 104 results
FAO: 34 results
World Bank: 31 results
Others: 38 results

Total - 2756

Study inclusion criteria

Included studies must have assessed schistosomiasis and water resources development of dams and irrigation schemes and compared the two as before and after construction and possibly included the associated snail control measures adopted. Factors used to identify suitable articles for inclusion were the availability and accuracy of data, ascertaining the observed changes in schistosomiasis prevalence and evaluating the adoption of engineering and biological snail control measures. Standard schistosomiasis control measures of mass drug administration (MDA)/chemotherapy with praziquantel, behavioural change, and health promotion campaigns were not included because they were out of the scope of this review. However, these measures were included in the recommendations as part of the overall integrated cross-sectoral approaches. Studies were not excluded according to year of publication. We reviewed the studies returned by the searches. First the titles were used to exclude papers not on schistosomiasis and water resources development of dams and irrigation schemes and snail control. Next, the abstracts were reviewed, and papers excluded according to the same criteria. These steps ensured that only data from suitable case studies were included.

Finally, the full texts were reviewed and papers excluded according to the aforementioned criteria. Papers were discussed between the reviewers and consensus was reached.

Data collection

Additional information on the included studies was extracted for country/region of study, year, article type/nature of evidence, and classified according to themes based on the definitions from inclusion criteria.

Quality assessment

We assessed included studies quality based on the nature of evidence, validity and journal, report or article reputation.

References

1. Tricco, AC, Lillie, E, Zarin, W, O'Brien, KK, Colquhoun, H, Levac, D, Moher, D, Peters, MD, Horsley, T, Weeks, L, Hempel, S et al. (2018) PRISMA extension for scoping reviews (PRISMA-ScR): checklist and explanation. Ann Intern Med. 2018,169(7):467-473. doi: 10.7326/M18-0850

2. Peters MDJ, Godfrey C, McInerney P, Munn Z, Tricco AC, Khalil, H. Scoping Reviews (2020). Aromataris E, Lockwood C, Porritt K, Pilla B, Jordan Z, editors. JBI Manual for Evidence Synthesis. JBI; 2024. Available from: https://synthesismanual.jbi.global. https://doi.org/10.46658/JBIMES-24-09
3. Steinmann, P., Keiser, J., Bos, R., Tanner, M., & Utzinger, J. (2006). Schistosomiasis and water resources development: systematic review, meta-analysis, and estimates of people at risk. The Lancet Infectious Diseases, 6(7), 411–425. https://doi.org/10.1016/S1473-3099(06)70521-7
4. Sokolow, S. H., Jones, I. J., Jocque, M., La, D., Cords, O., Knight, A., Lund, A., Wood, C. L., Lafferty, K. D., Hoover, C. M., Collender, P. A., Remais, J. V., Lopez-Carr, D., Fisk, J., Kuris, A. M., & De Leo, G. A. (2017). Nearly 400 million people are at higher risk of schistosomiasis because dams block the migration of snail-eating river prawns. Philosophical Transactions of the Royal Society B: Biological Sciences, 372(1722), 20160127. https://doi.org/10.1098/rstb.2016.0127
