# Supplementary material for: Schistosomiasis and water resources development in Africa: A scoping review and multi-case evaluation of associated snail control
Source: PLoS Negl Trop Dis. 2025 Jun 12;19(6):e0013180. doi: 10.1371/journal.pntd.0013180 (PMC12193731; doi:10.1371/journal.pntd.0013180)
Supplement: S1 Data — (DOCX) [file pntd.0013180.s006.docx]

Table S2: Method used to assess and classify non-relevant data for exclusion and papers for inclusion

| Read the title of the paper and classify it using column A |  |  |
| --- | --- | --- |
|  |  |  |
| **A: Title** |  | **Action** |
| Is this paper a duplicate of another | → YES | exclude |
| ↓NO |  |  |
| Is this paper not about schistosomiasis | → YES | exclude |
| ↓NO |  |  |
| Is this paper primarily about diagnostic test | → YES | exclude |
| ↓NO |  |  |
| Is this paper about treating or developing a vaccine against schistosomiasis | → YES | exclude |
| ↓NO |  |  |
| Is this paper about mass drug administration MDA | → YES | exclude |
| ↓NO |  |  |
| Is this paper not about human schistosomiasis infection | → YES | exclude |
| ↓NO |  |  |
| Is this paper about the effect of behaviour change or health education interventions on schistosomiasis | → YES | exclude |
| ↓NO |  |  |
| Read abstract and move to column B |  |  |
|  |  |  |
| Read the abstract of the paper and classify it using column B |  |  |
|  |  |  |
| **B: Abstract** |  | **Action** |
| Is this abstract not about impact of dams and irrigation schemes on schistosomiasis | → YES | exclude |
| ↓NO |  |  |
| Is this abstract primarily about environmental impact assessments of planned dams and irrigation schemes | → YES | exclude |
| ↓NO |  |  |
| Does this study not discuss schistosomiasis infection around dams and irrigation schemes | → YES | exclude |
| ↓NO |  |  |
| Read full paper and move to column C |  |  |
|  |  |  |
| **C: Full paper** |  |  |
| Excluded papers - ineligible study because no information about percentage infection | 25 |  |
| Excluded papers - non relevant, inseparable data because it includes other areas outside of dam location | 12 |  |
| Excluded papers - repeated data | 27 |  |
| Included papers with:   - % Infection reported - % increase in Schistosomiasis due to dam - Engineering and biological snail control measures adopted during/before/after construction of the dam - Monitoring/Evaluation | 122 |  |
